# Reducing Central Nervous System–Active Medications to Prevent Falls and Injuries Among Older Adults: A Cluster Randomized Clinical Trial

**DOI:** 10.1001/jamanetworkopen.2024.24234

**Published:** 2024-07-25

**Authors:** Elizabeth A. Phelan, Brian D. Williamson, Benjamin H. Balderson, Andrea J. Cook, Annalisa V. Piccorelli, Monica M. Fujii, Kanichi G. Nakata, Vina F. Graham, Mary Kay Theis, Justin P. Turner, Cara Tannenbaum, Shelly L. Gray

**Affiliations:** 1 Department of Medicine, Division of Gerontology and Geriatric Medicine, School of Medicine, University of Washington, Seattle; 2 Department of Health Systems and Population Health, School of Public Health, University of Washington, Seattle; 3 Kaiser Permanente Washington Health Research Institute, Kaiser Permanente Washington, Seattle; 4 Department of Biostatistics, University of Washington, Seattle; 5 Vaccine and Infectious Disease Division, Fred Hutchinson Cancer Center, Seattle, Washington; 6 Center for Medication Use and Safety, Faculty of Pharmacy and Pharmaceutical Sciences, Monash University, Melbourne, Australia; 7 Faculty of Medicine, University of Montreal, Montreal, Quebec, Canada; 8 Faculty of Pharmacy, University of Montreal, Montreal, Quebec, Canada; 9 Department of Pharmacy, School of Pharmacy, University of Washington, Seattle

## Abstract

**Importance:**

High-risk medications that contribute to adverse health outcomes are frequently prescribed to older adults. Deprescribing interventions reduce their use, but studies are often not designed to examine effects on patient-relevant health outcomes.

**Objective:**

To test the effect of a health system–embedded deprescribing intervention targeting older adults and their primary care clinicians for reducing the use of central nervous system–active drugs and preventing medically treated falls.

**Design, Setting, and Participants:**

In this cluster randomized, parallel-group, clinical trial, 18 primary care practices from an integrated health care delivery system in Washington state were recruited from April 1, 2021, to June 16, 2022, to participate, along with their eligible patients. Randomization occurred at the clinic level. Patients were community-dwelling adults aged 60 years or older, prescribed at least 1 medication from any of 5 targeted medication classes (opioids, sedative-hypnotics, skeletal muscle relaxants, tricyclic antidepressants, and first-generation antihistamines) for at least 3 consecutive months.

**Intervention:**

Patient education and clinician decision support. Control arm participants received usual care.

**Main Outcomes and Measures:**

The primary outcome was medically treated falls. Secondary outcomes included medication discontinuation, sustained medication discontinuation, and dose reduction of any and each target medication. Serious adverse drug withdrawal events involving opioids or sedative-hypnotics were the main safety outcome. Analyses were conducted using intent-to-treat analysis.

**Results:**

Among 2367 patient participants (mean [SD] age, 70.6 [7.6] years; 1488 women [63%]), the adjusted cumulative incidence rate of a first medically treated fall at 18 months was 0.33 (95% CI, 0.29-0.37) in the intervention group and 0.30 (95% CI, 0.27-0.34) in the usual care group (estimated adjusted hazard ratio, 1.11 (95% CI, 0.94-1.31) (*P* = .11). There were significant differences favoring the intervention group in discontinuation, sustained discontinuation, and dose reduction of tricyclic antidepressants at 6 months (discontinuation adjusted rate: intervention group, 0.23 [95% CI, 0.18-0.28] vs usual care group, 0.13 [95% CI, 0.09-0.17]; adjusted relative risk, 1.79 [95% CI, 1.29-2.50]; *P* = .001) and secondary time points (9, 12, and 15 months).

**Conclusions and Relevance:**

In this randomized clinical trial of a health system–embedded deprescribing intervention targeting community-dwelling older adults prescribed central nervous system–active medications and their primary care clinicians, the intervention was no more effective than usual care in reducing medically treated falls. For health systems that attend to deprescribing as part of routine clinical practice, additional interventions may confer modest benefits on prescribing without a measurable effect on clinical outcomes.

**Trial Registration:**

ClinicalTrials.gov Identifier: NCT05689554

## Key Points

**Question:**

Does a health system–embedded deprescribing intervention delivered to community-dwelling older adults and their primary care clinicians reduce use of central nervous system (CNS)–active medications and medically treated falls?

**Findings:**

In this cluster randomized, parallel-group clinical trial that included 2367 older adults and their primary care clinicians, medically treated falls were not reduced among those who received the intervention compared with usual care.

**Meaning:**

A deprescribing intervention focused on CNS-active medications that targeted older adults and their primary care clinicians was no more effective than usual care in improving the safety profile of medication regimens and averting serious fall events.

## Teaser

This cluster randomized clinical trial tests a health system–embedded deprescribing intervention targeting older adults and their primary care clinicians for reducing the use of central nervous system (CNS)–active drugs and preventing medically treated falls.

## Introduction

Medications that act on the central nervous system (CNS) are associated with cognitive impairment and falls among older people.^1,2,3,4^ One-fourth of community-dwelling older adults take 1 or more CNS-active medications.^5,6,7^ Although practice guidelines recommend that clinicians conduct periodic medication reviews to reduce use of these high-risk medications,^8^ many barriers preclude adherence to this recommendation.^9,10,11^

Promising approaches to curb medication use have engaged pharmacists and delivered direct-to-patient education on risks of CNS-active medications and safer alternatives and demonstrated medication reduction.^12,13,14^ However, interventions were focused on a limited number of classes of CNS-active medications, did not directly engage clinicians, and did not assess effects on preventable health outcomes, such as medically treated falls (ie, falls for which medical attention is sought).^15^ The STOP-FALLS (Reducing CNS-Active Medications to Prevent Falls and Injuries in Older Adults) trial^16^ was designed to test the effectiveness of a deprescribing intervention targeting community-dwelling older adults and their primary care clinicians to reduce use of CNS-active medications and prevent medically treated falls.

## Methods

### Trial Oversight

This was a pragmatic, cluster randomized clinical trial with a median follow-up of 24.8 months (IQR, 7.0-24.8 months). Details of the study methods have been described previously and the trial protocol has been published and registered on ClinicalTrials.gov (NCT05689554) (Supplement 1).^16^ The Kaiser Permanente Washington (KPWA) institutional review board approved the study. It granted a waiver of informed consent for eligible participants consistent with requirements outlined in 45 CFR 46.116 Part F.3, given the pragmatic and educational nature of the intervention and the high likelihood of differential refusals due to contact with intervention participants but not control participants and the resultant risk of bias in trial outcomes. The Consolidated Standards of Reporting Trials (CONSORT) reporting guideline was followed.

### Setting and Participants

Primary care clinics of the KPWA integrated group practice, along with their eligible patients, were recruited from April 1, 2021, to June 16, 2022. Eighteen clinics participated: 9 per study arm. Patients were eligible for enrollment if they were assigned to a primary care clinician or had 1 or more visits in the prior year with a clinician at 1 of the participating clinics and had evidence of chronic use of at least 1 of the 5 medication classes targeted by the intervention, defined as pharmacy dispensing of the medication for a minimum of 70 of the prior 90 days. Age eligibility was 60 years or older for opioids and sedative-hypnotics (benzodiazepines and “Z-drugs” [ie, zolpidem, zaleplon, zopiclone, and eszopiclone]) and 65 years or older for skeletal muscle relaxants, tricyclic antidepressants (TCAs), and first-generation antihistamines, consistent with KPWA guidelines. Information on race and ethnicity (Asian, Black or African American, Hispanic, Native Hawaiian or Pacific Islander, White, other race [unspecified], and multiple races) was collected. Race and ethnicity were determined based on patient self-report in the electronic medical record. Response options map to the National Institutes of Health racial and ethnic categories; the “other” category is a primarily collected (ie, not aggregated) category. Exclusion criteria were diagnosis of metastatic cancer, opioid use disorder, or dementia (or cholinesterase inhibitor or memantine prescription); inability to read print materials due to blindness; inability to read English; enrollment in any KPWA opioid-deprescribing study or health system initiative; receipt of palliative care or hospice services; or residence in a skilled nursing facility.

### Randomization

Randomization was at the clinic level to avoid cross-clinician contamination.^17^ Clinic pairs were matched on size (ie, number of patients of the target age group served) and geographic location.^18,19^ Randomization was computer generated and constrained randomization was used. Allocation concealment and implementation have been previously described.^16^ Intervention clinicians and participants were not blinded to group assignment.

### Study Groups

The intervention, modeled after that of D-PRESCRIBE (Developing Pharmacist-Led Research to Educate and Sensitize Community Residents to the Inappropriate Prescriptions Burden in the Elderly),^13^ was adapted in partnership with intervention clinics and systemwide primary care and pharmacy leadership. It consisted of patient education and clinician decision support to reduce use of the target medications. Patient education included theory-driven,^20^ medication-specific educational brochures; self-care handouts with tips for managing symptoms using nonpharmacologic approaches; and a public-domain brochure on ways to prevent falls. Materials were delivered via postal mail.^16^ Decision support included evidence-based pharmaceutical opinions (EBPOs) and deprescribing pearls (eAppendix in Supplement 2). The EBPOs, modeled after those used in a prior trial,^13^ addressed risks of the target medication class and evidence-based treatment alternatives and included hyperlinks to practice supports for deprescribing. A brief version of the EBPO was sent to clinicians via the electronic health record (EHR) staff messaging system with a hyperlink to the full-length EBPO on the study website. The EBPOs were delivered to the clinician synchronous with mailing of the patient education materials. Clinicians received an EBPO for each target medication class that their patient was prescribed.

Deprescribing pearls covered 13 different topics related to deprescribing along with “conversation starters” (short phrases to facilitate deprescribing discussions). The pearls were delivered via periodic emails to the clinic’s study champion (clinician volunteer or clinic director),^16^ who disseminated these materials as they saw fit (eg, via email).

The study arm receiving usual care (ie, care as ordinarily received in daily clinical practice) did not receive the STOP-FALLS intervention and was unaware of the study.

### Primary Outcome

The primary outcome was time (in days) to the first medically treated fall, ascertained from electronic utilization files of the health plan, which capture all health care encounters for patients in KPWA’s integrated group practice. *International Statistical Classification of Diseases and Related Health Problems, Tenth Revision* (*ICD-10*), injury codes (S or T), musculoskeletal disease codes (M), or fall-related cause of injury codes (W) were used to identify medically treated falls.^16,21^

### Secondary Outcomes

Secondary outcomes assessed changes to target medications at 6, 9, 12, and 15 months of follow-up, including discontinuation (defined as no prescription fill for 90 days), sustained discontinuation (defined as no prescription fill for 180 days), and dose reduction (defined as mean standardized daily dose [SDD] for 90 days after the time point of interest minus baseline SDD).^22^ We considered each target medication separately and summarized across all target medication classes (defined relative to the first mailed medication brochure and referred to as “first target medication”). Drug name, dose, frequency, route, and days’ supply were obtained from automated pharmacy records of the health plan to assess these outcomes. The 6-month time point was chosen as the primary end point for medication outcomes based on prior deprescribing studies.^13,14^

### Safety Assessments

Safety assessments included death, serious adverse drug withdrawal events (ADWEs) related to opioids or benzodiazepines, and unintentional overdose. Death data were obtained from KPWA automated data files. An ADWE was defined as an urgent care or emergency department visit or hospitalization based on *ICD-10* codes for symptoms related to drug withdrawal and ascertained from electronic health plan utilization files (eTable 1 in Supplement 2). Two study team members (E.A.P. and S.L.G.) independently reviewed the abstracted medical record, blinded to intervention status, and used a published algorithm^23^ to assess the probability that symptoms represented an ADWE. Adverse drug withdrawal events scored as possible, probable, or definite are reported herein. Unintentional overdoses were identified using *ICD-10* codes from a Centers for Disease Control and Prevention coding schema for national overdose surveillance (eTable 2 in Supplement 2).^24^

### Other Outcomes

Plans for tapering target medications were ascertained from *signetur* (patient instruction) fields of prescription medications in the EHR using methods developed previously.^25^ Willingness to deprescribe was assessed by a 2-item survey adapted from a prior trial^14^ and mailed anonymously after delivery of an educational brochure. The items were (1) the brochure will help prepare me to talk with my clinician, and (2) I will start a conversation about tapering.

### Sample Size Calculation

The sample size for the primary outcome was based on a test of the relative risk, using PASS 2019 software, version 19.0.1 (NCSS Statistical Software).^26^ This minor simplification based on a binary outcome, after inflating the sample size by 15%, is a conservative approach to a calculation based on the marginal hazard ratio.^27^ We estimated that a sample size of 18 clinics would provide 89% power to detect a 20% reduction in the rate of medically treated falls in the intervention group vs usual care, assuming 18 months’ follow-up for all participants, an equal number of clinics assigned to each trial arm, annual loss to follow-up rate of 5%, annual death rate (ie, competing risk) of 5%, and an intracluster correlation coefficient of 0.001 estimated from a historical cohort of potentially eligible participants at participating KPWA clinics.

### Statistical Analysis

All analyses were intention to treat. The primary outcome was analyzed using methods for time-to-event outcomes that account for both competing risks and cluster randomization.^16,28,29^ We used a small-cluster correction factor equal to 17 (18 clusters − 1 cluster-level randomization factor).^30^ We used a cause-specific proportional hazards regression model to estimate an adjusted cause-specific hazard ratio and corresponding CI and *P* value using the R package survival (R Project for Statistical Computing); these formed the basis for inference on the intervention effect.^31,32,33^ To support this analysis, we estimated the adjusted cause-specific hazard ratio of death and adjusted cause-specific cumulative incidence curves for the first incident medically treated fall and death using the R package timereg.^34,35^ In a sensitivity analysis, we considered a composite outcome of time to first incident medically treated fall or death and used a proportional hazards model to estimate an adjusted hazard ratio. We examined heterogeneity of the intervention effect on time to first incident medically treated fall in 5 prespecified subgroups: age (<80 vs ≥80 years),^36^ sex assigned at birth (female vs male), at least 1 fall prior to the first brochure mailing date, multimorbidity (<2 vs ≥2 chronic conditions), and frailty. In all models, we adjusted for clinic geographic region, age, sex, and an indicator of prior falls (defined as a fall within 90 days prior to baseline). We obtained 95% CIs for all point estimates, and obtained these estimates overall and at 6, 9, and 12 months.

Discontinuation and sustained discontinuation of each target medication at 6, 9, 12, and 15 months (discontinuation only) were analyzed using a modified Poisson regression model^37^ fitted using generalized estimating equations (using the R package gee)^38^ adjusted for baseline SDD of the given medication. We estimated the adjusted relative risk of medication discontinuation for each medication class comparing the adjusted rates in the intervention group and the usual care group. Dose reduction at 6, 9, 12, and 15 months was analyzed using generalized estimating equations linear regression (using the R package geepack)^39^ adjusted for the baseline dose of the given medication and the same variables as the primary outcome analysis, and separate models were fitted for each time point. Follow-up time was included as an offset term in the Poisson regression models and as a weight in the linear regression models. Generalized estimating equation models were fitted in all analyses to account for correlation due to clinic randomization, and in all cases we used a correction for the small number of clusters.^30^

For the safety outcomes of death, serious ADWEs, and unintentional overdoses, we report rates in both the intervention and usual care groups. Tapering plans were described by computing the number of participants with a taper documented for each target medication over 6 months after the medication-specific brochure mailing. Participant willingness to deprescribe was analyzed as the percentage of agreement with each survey item. All *P* values were from 2-sided tests and results were deemed statistically significant at *P* < .05. Statistical analyses were performed using R software, version 4.0.2.

## Results

### Participants

From April 1, 2021, to June 16, 2022, we randomized 18 clinics to participate in the trial. Among patients of these clinics who met the trial’s eligibility criteria, 1235 were excluded due to having died or disenrolled from the health plan or no longer having a prescription for one of the target medications prior to first mailing of intervention materials. There were 159 of 1106 participants in the intervention group (14%) and 206 of 1261 participants in the usual care group (16%) who were censored due to disenrollment from the health plan prior to having a medically treated fall. A total of 72 of 1106 participants (7%) in the intervention group and 86 of 1261 (7%) in the usual care group died. Health plan disenrollment and death were the only reasons for loss to follow-up. Outcome analyses are based on 2367 patients (Figure).

**Figure.**
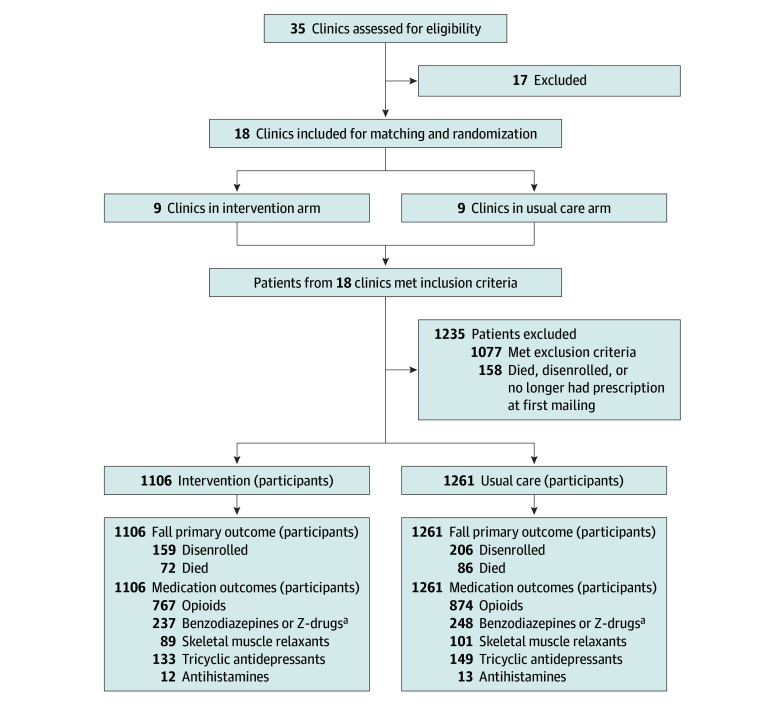
Participant Flow in the STOP-FALLS (Reducing CNS [Central Nervous System]–Active Medications to Prevent Falls and Injuries in Older Adults) Trial ^a^Z-drugs include the sedative-hypnotics zolpidem, zaleplon, zopiclone, and eszopiclone.

Demographic and health characteristics are shown in Table 1. The intervention and usual care arms were comparable at baseline. Across the groups, the mean (SD) age was 70.6 (7.6) years; 1488 patients (63%) were female and 879 (37%) were male; 37 patients (2%) were Asian, 68 (3%) were Black or African American, 78 (3%) were Hispanic, 12 (1%) were Native Hawaiian or Pacific Islander, 2056 (87%) were White, 35 (2%) were other race, and 56 (2%) were multiple races. The mean (SD) number of chronic conditions was 2.2 (1.5). A total of 81 patients (3%) were frail^40^ and 677 patients (29%) had sought medical care for a fall in the prior year. Most patients were prescribed 1 CNS-active medication targeted by the intervention; opioids (1625 [69%]), benzodiazepines (321 [14%]), and TCAs (268 [11%]) were the most commonly prescribed medications.

**Table 1.** Baseline Characteristics of STOP-FALLS Participants

Characteristic	Participants, No. (%)		
	Overall (N = 2367)	Intervention arm (n = 1106)	Usual care arm (n = 1261)
**Demographic variables**			
Age, mean (SD), y	70.6 (7.6)	70.3 (7.4)	70.9 (7.7)
Age group, y			
60-64	527 (22)	245 (22)	282 (22)
65-74	1224 (52)	594 (54)	630 (50)
75-84	487 (21)	217 (20)	270 (21)
≥85	129 (5)	50 (5)	79 (6)
Sex			
Female	1488 (63)	704 (64)	784 (62)
Male	879 (37)	402 (36)	477 (38)
Self-reported race and ethnicity			
American Indian or Alaska Native	29 (1)	18 (2)	11 (1)
Asian	37 (2)	16 (1)	21 (2)
Black or African American	68 (3)	38 (3)	30 (2)
Hispanic	78 (3)	38 (3)	40 (3)
Native Hawaiian or Pacific Islander	12 (1)	8 (1)	4 (0.3)
White	2056 (87)	957 (87)	1099 (87)
Other race^a^	35 (2)	10 (1)	25 (2)
Multiple races	56 (2)	24 (2)	32 (3)
Unknown or not reported	74 (3)	35 (3)	39 (3)
**Health status variables**			
Use of mobility aid	31 (1)	12 (1)	19 (2)
Difficulty walking	22 (1)	8 (1)	14 (1)
Orthostatic hypotension	21 (1)	12 (1)	9 (1)
No. of chronic conditions, mean (SD)	2.2 (1.5)	2.3 (1.6)	2.2 (1.5)
History of stroke	91 (4)	37 (3)	54 (4)
History of fracture	43 (2)	21 (2)	22 (2)
Frailty status^b^^,^^c^			
Nonfrail	1071 (45)	501 (45)	570 (45)
Prefrail	1215 (51)	565 (51)	650 (52)
Frail	81 (3)	40 (4)	41 (3)
Legal blindness	2 (<0.1)	1 (<0.1)	1 (<0.1)
Musculoskeletal pain	1338 (57)	612 (55)	726 (58)
Hearing loss	132 (6)	62 (6)	70 (6)
Cognitive impairment	20 (1)	9 (1)	11 (1)
Alcohol use disorder	32 (1)	14 (1)	18 (1)
Medical care for a fall in past 12 mo	677 (29)	328 (30)	349 (28)
**Medication variables**			
Medication class targeted by the intervention			
Opioid	1625 (69)	761 (69)	864 (69)
Benzodiazepine	321 (14)	153 (14)	168 (13)
Z-drug^d^	177 (8)	92 (8)	85 (7)
Tricyclic antidepressant	268 (11)	123 (11)	145 (11)
Muscle relaxant	180 (8)	85 (8)	95 (8)
Antihistamine (prescription)	53 (2)	23 (2)	30 (2)
1 Target medication	2127 (90)	982 (89)	1145 (91)
≥2 Target medications	257 (10)	131 (11)	116 (9)
Other medication class			
Antidepressant	1107 (47)	524 (47)	583 (46)
Antiseizure drugs	156 (7)	73 (7)	83 (7)
Gabapentinoid	645 (27)	309 (28)	336 (27)
Urinary antispasmodic	113 (5)	50 (5)	63 (5)
Antihypertensive	776 (33)	362 (33)	414 (33)

Abbreviation: STOP-FALLS, Reducing CNS (Central Nervous System)–Active Medications to Prevent Falls and Injuries in Older Adults.

^a^ Other race as reported by the participant in their electronic medical record field (primarily unspecified).

^b^ Frailty assessed using the Claims-Based Frailty Index.^40^

^c^ Percentages may not total 100 due to rounding.

^d^ Z-drugs include the sedative-hypnotics zolpidem, zaleplon, zopiclone, and eszopiclone.

### Primary Outcome

The adjusted cumulative incidence at 18 months of a first medically treated fall was 33% in the intervention group (adjusted cumulative incidence rate, 0.33 [95% CI, 0.29-0.37]) and 30% in the usual care group (adjusted cumulative incidence rate, 0.30 [95% CI, 0.27-0.34]) (Table 2). The adjusted cause-specific instantaneous risk of a first medically treated fall among those who had neither fallen nor died at any given time point was 1.11 times higher in the intervention group than in the usual care group (estimated hazard ratio, 1.11 [95% CI, 0.94-1.31]; *P* = .11). Time to first medically treated fall was not significantly different between the study arms at any time point. The adjusted cumulative incidence of death at 18 months was similar across arms (0.02 [95% CI, 0.01-0.03] in the intervention arm and 0.02 [95% CI, 0.01-0.03] in the usual care arm) and was not significantly different between the study arms at any time point. Results of the composite outcome analysis were similar. We observed no significant heterogeneity of the intervention effect on medically treated falls or death among prespecified subgroups (eTable 3 in Supplement 2).

**Table 2.** Primary Outcomes

Outcome	Adjusted cumulative incidence rate (95% CI)^a^		Cause-specific outcome analysis^b^	
	Usual care (n = 1261)	Intervention (n = 1106)	Adjusted HR (95% CI)^c^	*P* value from LRT
**Primary analysis**				
Time to first medically treated fall or fall-related death				
Overall^d^	0.30 (0.27-0.34)	0.33 (0.29-0.37)	1.11 (0.94-1.31)	.11
6 mo	0.14 (0.13-0.16)	0.16 (0.14-0.18)	1.14 (0.95-1.36)	.17
9 mo	0.19 (0.17-0.21)	0.20 (0.18-0.23)	1.09 (0.90-1.32)	.29
12 mo	0.23 (0.20-0.26)	0.25 (0.22-0.29)	1.08 (0.90-1.29)	.34
Time to non–fall-related death (competing risk of primary outcome)				
Overall^d^	0.02 (0.01-0.03)	0.02 (0.01-0.03)	0.83 (0.52-1.33)	.44
6 mo	0.01 (0.00-0.01)	0.01 (0.00-0.01)	0.42 (0.19-0.90)	.08
9 mo	0.01 (0.00-0.02)	0.01 (0.00-0.01)	0.44 (0.20-0.98)	.06
12 mo	0.01 (0.00-0.02)	0.01 (0.00-0.02)	0.64 (0.34-1.21)	.21
**Secondary analysis**				
Time to composite outcome of first medically treated fall or death				
Overall^d^	0.33 (0.29-0.37)	0.35 (0.33-0.39)	1.09 (0.92-1.29)	.18
6 mo	0.15 (0.13-0.17)	0.16 (0.14-0.18)	1.10 (0.91-1.33)	.30
9 mo	0.20 (0.17-0.23)	0.22 (0.19-0.24)	1.06 (0.86-1.29)	.50
12 mo	0.25 (0.22-0.28)	0.27 (0.24-0.30)	1.05 (0.87-1.27)	.50

Abbreviations: HR, hazard ratio; LRT, log-rank test.

^a^ Adjusted cumulative incidence rates are estimated using a competing risk model for the 2 competing risk outcomes, time to first medically treated fall and time to non–fall-related death, using a subdistribution hazards model to estimate adjusted cause-specific cumulative incidences of both first medically treated fall and death over time, accounting for clustering due to clinic randomization using robust SEs. A non–fall-related death is defined as death with no preceding fall care. Time is measured in days. For the composite secondary outcome, time to first medically treated fall or death, we used a Cox proportional hazards regression model with a Nelson-Aalen estimator of the cumulative incidence. All models censored at time of disenrollment and adjusted cumulative incidence are calculated using the mean of the overall covariate distribution. All models adjusted for geographic region of the clinic, age, sex assigned at birth, and any falls prior to baseline.

^b^ Adjusted cause-specific outcome analysis results are obtained using a cause-specific Cox proportional hazards regression model for a given outcome, censoring for disenrollment, study follow-up end, or for the competing risk outcome if applicable (eg, for time to medically treated fall the analysis would censor for death). For 6, 9, and 12 months, censoring will also occur at the time point after the enrollment date. All models adjusted for geographic region of the clinic, age, sex assigned at birth, and any falls prior to baseline.

^c^ The 95% CIs are Wald type. In cases with a small number of events, this can lead to discrepancies in inference between the 95% CI and a *P* value based on a log-rank test.

^d^ Overall estimates are cumulative incidence rates at 18 months, because most participants had at least 18 months of follow-up, but uses all data available for a given participant for the analysis.

### Secondary Outcomes

Secondary (medication) outcomes at 6 months (primary end point) are shown in Table 3 and Table 4. There were significant differences favoring the intervention group in discontinuation, sustained discontinuation, and dose reduction for TCAs (discontinuation adjusted rate: intervention group, 0.23 [95% CI, 0.18-0.28] vs usual care group, 0.13 [95% CI, 0.09-0.17]; adjusted relative risk, 1.79 [95% CI, 1.29-2.50]; *P* = .001) but not for other medication classes.

**Table 3.** Binary Medication Outcomes After 6 Months From Mailing for a Given Medication

Binary outcome	Analytic sample, No.	Adjusted incidence rate (95% CI)^a^		Adjusted RR (95% CI)^a^	*P* value
		Usual care (n = 1261)	Intervention (n = 1106)		
**Discontinuation (90-d SDD = 0 after 6 mo)**					
Opioid	1588	0.04 (0.03-0.06)	0.04 (0.03-0.06)	0.88 (0.54-1.45)	.63
Benzodiazepine or Z-drug^b^	465	0.20 (0.17-0.25)	0.20 (0.17-0.25)	0.99 (0.78-1.26)	.94
Tricyclic antidepressant	273	0.13 (0.09-0.17)	0.23 (0.18-0.28)	1.79 (1.29-2.50)	.001
Muscle relaxant	184	0.39 (0.33-0.47)	0.39 (0.27-0.57)	0.99 (0.65-1.51)	.97
Antihistamine (prescription)	55^c^	Convergence not achieved	NA	NA	NA
First target medication^d^	2288	0.09 (0.08-0.11)	0.12 (0.09-0.15)	1.24 (0.90-1.70)	.18
**Sustained discontinuation (180-d SDD = 0 after 6 mo)**					
Opioid	1588	0.04 (0.03-0.06)	0.03 (0.02-0.05)	0.83 (0.50-1.38)	.47
Benzodiazepine or Z-drug^b^	465	0.19 (0.16-0.23)	0.20 (0.17-0.24)	1.05 (0.81-1.36)	.71
Tricyclic antidepressant	273	0.10 (0.07-0.15)	0.19 (0.15-0.24)	1.80 (1.26-2.58)	.001
Muscle relaxant	184	0.37 (0.30-0.46)	0.37 (0.25-0.53)	0.99 (0.64-1.53)	.96
Antihistamine (prescription)	55^e^	Convergence not achieved	NA	NA	NA
First target medication^d^	2288	0.08 (0.07-0.10)	0.11 (0.08-0.14)	1.25 (0.91-1.74)	.17

Abbreviations: NA, not applicable; RR, relative risk; SDD, standardized daily dose.

^a^ Adjusted rates, relative risks, and corresponding 95% CIs and *P* values are calculated from a Poisson regression model for the given binary outcome with an offset for proportion of days enrolled within outcome window at 6 months (90 days for discontinuation and 180 days for sustained discontinuation). Model fitted using generalized estimating equations to account for correlation due to clinic randomization using a small number of cluster correction. All analyses adjusted for baseline SDD dose of the given medication analyzed, age, sex assigned at birth, geographic region of the clinic, and any falls prior to baseline. Adjusted rates are calculated at the population mean level of the covariate.

^b^ Z-drugs include the sedative-hypnotics zolpidem, zaleplon, zopiclone, and eszopiclone.

^c^ A total of 5 patients discontinued use at 90 days after 6 months.

^d^ First target medication is the first medication mailed to the participant at the time of study enrollment.

^e^ A total of 5 patients sustained discontinuation 180 days after 6 months.

**Table 4.** Continous Medication Outcomes After 6 Months From Mailing for a Given Medication

Continuous outcome	Analytic sample, No.	Adjusted mean (95% CI)^a^		Adjusted difference (95% CI)^a^	*P* value^a^
		Usual care (n = 1261)	Intervention (n = 1106)		
Dose reduction^b^					
Opioid	1588	−0.28 (−0.36 to 0.20)	−0.29 (−0.38 to 0.21)	−0.01 (−0.13 to 0.11)	.86
Benzodiazepine or Z-drug^c^	465	−0.91 (−1.13 to 0.69)	−0.84 (−1.00 to 0.68)	0.07 (−0.22 to 0.36)	.62
Tricyclic antidepressant	273	−0.72 (−1.15 to 0.29)	−1.57 (−1.94 to 1.20)	−0.85 (−1.35 to 0.34)	.001
Muscle relaxant	184	−2.73 (−3.14 to 2.31)	−2.66 (−3.3 to 2.03)	0.06 (−0.72 to 0.85)	.88
Antihistamine (prescription)	55	−0.10 (−0.19 to 0.01)	−0.13 (−0.27 to 0.01)	−0.03 (−0.19 to 0.13)	.71
First target medication	2288	−0.47 (−0.61 to 0.32)	−0.61 (−0.68 to 0.54)	−0.14 (−0.29 to 0.01)	.06

^a^ Adjusted means, mean differences, and corresponding 95% CIs and *P* values are calculated from a weighted regression model for the continuous outcome dose reduction, with the weight being number of days enrolled in the 90-day outcome window at 6 months fitted using generalized estimating equations to account for correlation due to clinic randomization using a small number of cluster correction. All analyses adjusted for baseline standardized daily dose of the given medication analyzed, age, sex assigned at birth, geographic region of the clinic, and any falls prior to baseline. Adjusted means are calculated at the population mean level of the covariate.

^b^ Dose reduction is defined as the change in medication dose at 6 months (mean standardized daily dose over 90 days after 6 months) minus baseline dose (mean standardized daily dose over 90 days before baseline).

^c^ Z-drugs include the sedative-hypnotics zolpidem, zaleplon, zopiclone, and eszopiclone.

Medication outcomes at secondary time points are shown in eTables 4, 5, and 6 in Supplement 2. Discontinuation of TCAs was significantly higher in the intervention compared with the usual care group at all follow-up time points. Discontinuation of sedative-hypnotics was significantly higher in the intervention group at 9 months. Discontinuation of antihistamines was significantly higher in the intervention group at 9 and 12 months. Discontinuation of the first targeted medication was significantly higher at 9 months. Sedative-hypnotic discontinuation rates in the usual care arm ranged from 20% (adjusted rate, 0.20, 95% CI, 0.17-0.25) at 6 months to 46% (adjusted rate, 0.46, 95% CI, 0.42-0.50) at 15 months. Skeletal muscle relaxant discontinuation rates in the usual care arm ranged from 39% (adjusted rate, 0.39, 95% CI, 0.33-0.47) at 6 months to 49% (adjusted rate, 0.49, 95% CI, 0.36-0.68) at 15 months.

### Safety Assessments (Death, Serious ADWE, and Unintentional Overdose)

We observed 86 deaths (7%) among the 1261 patients in the usual care group and 72 deaths (7%) among the 1106 patients in the intervention group over the course of the study. Among those using a benzodiazepine or an opioid, ADWEs occurred more frequently in the intervention group compared with the usual care group (10 of 914 [1%] vs 4 of 1032 events [0.4%]). All events were scored as “possible,” except for 1 event that was scored as “probable,” with the latter event occurring in the intervention arm. Unintentional overdoses were rare, with 11 total such episodes overall, 5 events among the 1261 patients (0.4%) in the usual care arm and 6 among the 1106 patients (1%) in the intervention arm.

### Other Outcomes

#### Medication Taper Plans

Nine participants in the intervention arm (7 prescribed an opioid, 2 a sedative-hypnotic) and no usual care participants had patient instructions indicating a taper of the target medication after the corresponding medication brochure was mailed.

#### Patient Willingness to Deprescribe

A total of 473 completed surveys were returned. More than half the respondents (243 [51%]) had received an opioid brochure; fewer than 20% of those (43 of 243 [18%]) agreed with each item. The percentage of agreement was higher for the other medication classes, ranging from 30% to 40% across the classes.

## Discussion

This cluster randomized trial found that a health system–embedded deprescribing intervention delivered to community-dwelling older adults with chronic prescribed use of 1 or more CNS-active medications and their primary care clinicians did not significantly reduce medically treated falls compared with usual care. High rates of deprescribing were observed in both arms for sedative-hypnotics, TCAs, and skeletal muscle relaxants. Significant differences in discontinuation, sustained discontinuation, and dose reduction over the entire follow-up period favoring the intervention arm occurred only for TCAs, although significant differences in discontinuation favoring the intervention arm were observed at 1 or more follow-up time points for sedative-hypnotics and antihistamines. Rates of death and unintentional overdose were comparable across study arms. The frequency of ADWEs was slightly higher in the intervention arm; however, these events were rare overall.

Trial results can be explained by similar rates of deprescribing in the intervention and usual care arms. Discontinuation rates of sedative hypnotics and muscle relaxants in the usual care arm were high (>40% at 15 months) and, other than for TCAs, similar between groups during 15 months of follow-up. Discontinuation rates for sedative-hypnotics in the usual care groups in other deprescribing trials have ranged from 5% to 26% at 6 months.^12,13,14^

Several deprescribing initiatives implemented by the delivery system contemporaneous with the STOP-FALLS trial may account for the high deprescribing rates in the usual care arm. These initiatives included a chronic pain clinic, where people prescribed high-dose opioids could be referred for intervention, an EHR “stop” that prohibited coprescribing of opioids and benzodiazepines, and an updated KPWA guideline on sedative-hypnotic prescribing.

Ours may be the first deprescribing trial to document a reduction in TCAs. System-level initiatives focusing on TCAs were not in place during the intervention period, which may explain why an effect of the intervention on TCAs was observed. The consistent pattern of higher TCA discontinuation rates in the intervention arm suggests that the intervention influenced clinicians to focus on this class of medications for their deprescribing efforts. Others have found that passive clinical decision support positively influences prescribing of potentially inappropriate medications.^41^ A search of the literature identified no trials of opioid deprescribing involving older adults; thus, future research should target this class of medications.

### Strengths and Limitations

This study has some strengths, including its rigorous design and outcome assessments derived from electronic data sources. Additional strengths include the complete capture of health care utilization, long duration (24 months) of follow-up for ascertainment of the outcomes, and sufficient power to detect an effect on medically treated falls. Results are generalizable to older people without diagnosed dementia who are not receiving palliative care.

This study also has some limitations, including the small number of participants of racial and ethnic minority groups, which reflects the underlying racial and ethnic makeup of the health care system and the geographic area. Participants were also primarily nonfrail. Because of the focus on pragmatically ascertainable outcomes, the effect of the intervention on outcomes such as nonprescription antihistamine use and non–medically treated falls is unknown. Last, the trial was conducted during the COVID-19 pandemic, during which outpatient visits were frequently conducted remotely; this factor, along with the additional demands on health care professionals posed by COVID-19, may have limited deprescribing considerations.

## Conclusions

This cluster randomized clinical trial found that a health system–embedded deprescribing intervention was no more effective than usual care in reducing medically treated falls among community-dwelling older adults prescribed CNS-active medications. For health systems that attend to deprescribing as part of routine clinical practice, additional interventions may confer modest benefits on prescribing without a measurable effect on clinical outcomes.

## Notes

Supplement 1.
Trial Protocol

Supplement 2. **eTable 1.** Adverse Drug Withdrawal Event Codes **eTable 2.** Unintentional Overdose Codes **eTable 3.** Heterogeneity of Treatment Effect for Time to First Medically Treated Fall and Non-Fall Death **eTable 4.** Summary of Medication Outcomes After 9 Months From Mailing for a Given Medication **eTable 5.** Summary of Medication Outcomes After 12 Months From Mailing for a Given Medication **eTable 6.** Summary of Medication Outcomes After 15 Months From Mailing for a Given Medication **eAppendix.** Evidence-Based Pharmaceutical Opinions

Supplement 3.
Data Sharing Statement
