# Supplementary material for: Reducing Central Nervous System–Active Medications to Prevent Falls and Injuries Among Older Adults: A Cluster Randomized Clinical Trial
Source: JAMA Netw Open. 2024 Jul 25;7(7):e2424234. doi: 10.1001/jamanetworkopen.2024.24234 (PMC11273227; doi:10.1001/jamanetworkopen.2024.24234)
Supplement: Supplement 1. — Trial Protocol [file jamanetwopen-e2424234-s001.pdf]

REDUCING CNS-ACTIVE MEDICATIONS TO PREVENT FALLS AND INJURIES IN OLDER ADULTS (STOP-FALLS)

Principal Investigators:
Elizabeth A. Phelan, MD, MS
Professor, Medicine/Gerontology and Geriatric Medicine, School of Medicine
Adjunct Professor, Health Systems and Population Health, School of Public Health
University of Washington

Shelly L. Gray, PharmD, MS
Professor, Elmer and Joy Plein Endowed Director of the Plein Center for Geriatric Pharmacy Research, Education and Outreach
University of Washington

Kaiser Permanente Washington Site Principal Investigator:
Benjamin Balderson, PhD
Senior Collaborative Scientist
Kaiser Permanente Washington Health Research Institute
Kaiser Permanente Washington

Supported by:

Centers for Disease Control and Prevention
GRANT NUMBER U01CE002967

TRIAL PROTOCOL
January 18, 2023

Contents

1 STUDY OBJECTIVES 4
1.1 Primary Objective 4
1.2 Secondary Objective 4
2 BACKGROUND AND RATIONALE 4
2.1 Background on Condition, Disease or Other Primary Study Focus 4
2.2 Study Rationale 5
3 STUDY DESIGN 5
4 SELECTION AND ENROLLMENT OF PARTICIPANTS 6
4.1 Inclusion Criteria 6
4.2 Exclusion Criteria 7
4.3 Study Enrollment Procedures 7
5 STUDY INTERVENTIONS 8
5.1 Interventions, Administration and Duration 8
5.2 Handling of Study Interventions 11
5.3 Concomitant Interventions 11
5.4 Adherence Assessment 11
6 STUDY PROCEDURES 12
6.1 Schedule of Evaluations 12
7 SAFETY ASSESSMENTS 12
7.1 Specifications of Safety Parameters 12
7.2 Methods and Timing for Assessing, Recording and Analyzing Safety Parameters 12
7.3 Adverse Events (AE) and Serious Adverse Events (SAE) 13
7.3.1 Reporting Procedures 13
7.3.2 Follow-up for Adverse Events 14
7.4 Safety Monitoring 14
8 INTERVENTION DISCONTINUATION 14

9 STATISTICAL CONSIDERATIONS 14
9.1 General Design Issues 14
9.2 Sample Size and Randomization 15
9.2.1 Treatment Assignment Procedures 15
9.3 Interim analyses and Stopping Rules 16
9.4 Outcomes 16
9.4.1 Primary outcome 16
9.4.2 Secondary outcomes 16
9.5 Data Analyses 17
10 DATA COLLECTION AND QUALITY ASSURANCE 19
10.1 Data Collection Sources 19
10.2 Data Management 20
11 PARTICIPANT RIGHTS AND CONFIDENTIALITY 20
11.1 Institutional Review Board (IRB) Review 20
11.2 Informed Consent 20
11.3 Participant Confidentiality 21
11.4 Study Discontinuation 21
12 ETHICAL CONSIDERATIONS 21
13 PUBLICATION OF RESEARCH FINDINGS 21
14 REFERENCES 22

1 STUDY OBJECTIVES

1.1 Primary Objective

The primary objective of this trial is to test the effectiveness of a health-system-embedded deprescribing intervention on the incidence of medically treated falls with a sample of older adults who are long-term users of one or more CNS-active medications. CNS-active medication classes targeted by the intervention include: opioids, sedative-hypnotics (benzodiazepines and Z-drugs), skeletal muscle relaxants, tricyclic antidepressants, and first-generation antihistamines. Participants will be followed for up to 26 months.

1.2 Secondary Objective

We will also examine 1) discontinuation or dose reductions of target medication (secondary outcomes); 2) serious adverse drug withdrawal events (ADWE), 3) unintentional overdose, and 4) death (safety outcomes); 5) evidence of planned dose reductions (process outcome) and 6) factors affecting intervention implementation.

2 BACKGROUND AND RATIONALE

2.1 Background on Condition, Disease or Other Primary Study Focus

Falls are the most frequent cause of fatal and non-fatal injuries among people aged 65 years and older (1). Falls and their associated injuries have multiple serious adverse consequences—avoidable emergency department (ED) visits and hospitalizations, loss of independence, decline in physical function, nursing home placement, and reduced quality of life (2-5). Of particular concern, national data from several countries indicate an alarming rise in fall-related ED visits, (6) hospitalizations, and injury care costs over the last decade (7). Thus, health systems approaches to prevent falls and fall injuries are urgently needed in order to "turn the tide."

Medication use, particularly use of medications that affect the central nervous system (CNS), has been consistently linked to falls (8-10). Common side effects of CNS-active medications include dizziness, sleepiness, and impaired balance and coordination. Use of CNS-active medications is common, with up to one-quarter of older adults in the community taking at least one of these medications (11).

Practice guidelines recommend that prescribers review all medications with their older patients to minimize polypharmacy and the use of CNS-active and other high-risk medications (12). However, this practice is not routinely followed (13-15) due to multiple

barriers, including lack of healthcare provider and patient awareness that medications can cause falls, (16) patients' belief in the need for medication, (17, 18) and provider reluctance to change prescriptions, even in the face of patients prompting the discussion (19).

The D-PRESCRIBE trial, a cluster randomized trial in Canada delivered by community pharmacists, evaluated an educational intervention directed to patients and provider decision support. The intervention was highly effective in reducing use of potentially inappropriate medications by older adults, including CNS-active medications (benzodiazepines and non-benzodiazepine hypnotics) (20). However, effects on health outcomes, including falls, were not reported. STOP-FALLS will adapt the D-PRESCRIBE intervention for use in an integrated healthcare delivery system in the United States and assess its effectiveness on medically treated falls.

2.2 Study Rationale

STOP-FALLS seeks to address the guideline-practice gap described under 2.1., above. Our research team adapted a set of best practices for reducing use of CNS-active medications in older adults by delivering a pragmatic, cluster-randomized trial in primary care clinics of Kaiser Permanente Washington (KPWA). The intervention is designed to activate patients and providers. It delivers direct-to-patient education and provider decision support about the risks of five classes of CNS-active medications: opioids, sedative-hypnotics, skeletal muscle relaxants, tricyclic antidepressants, and first-generation antihistamines.

3 STUDY DESIGN

This is a pragmatic, cluster-randomized, parallel-group, controlled clinical trial. The unit of randomization is the clinic, to avoid the risk of contamination if healthcare providers within a clinic were randomized (i.e., reducing the potential for intervention providers to communicate with control providers about the intervention and share materials). Eighteen clinics will be identified for the trial, of which 9 will be randomized to the intervention and 9 to usual care. **Figure 1** (below) illustrates the study design and flow.

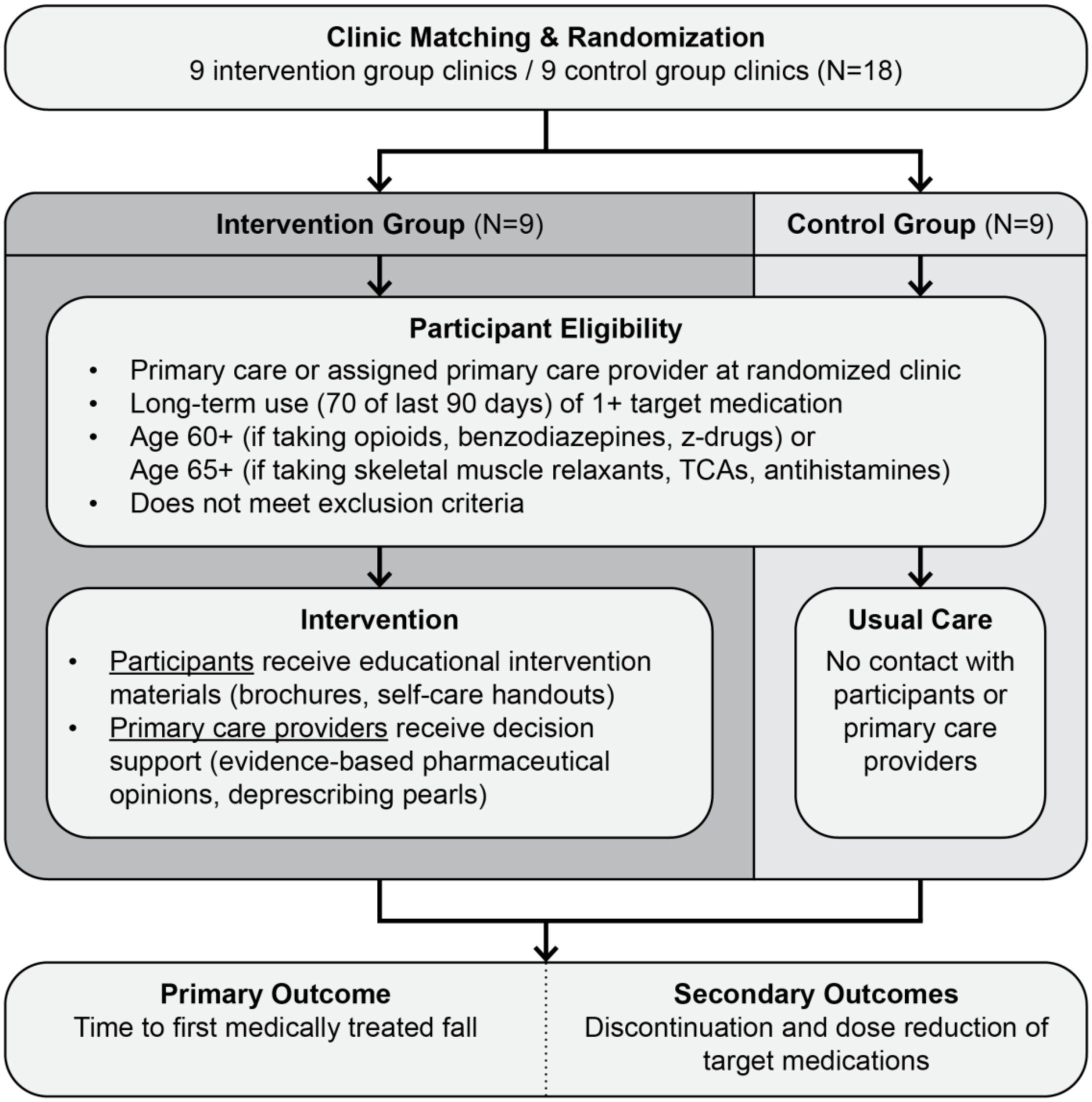

4 SELECTION AND ENROLLMENT OF PARTICIPANTS

4.1 Inclusion Criteria

Clinic Eligibility

From among the 35 KPWA primary care clinics, we will identify a subset of clinics to represent a diverse geographical range, excluding clinics that serve a small eligible patient population to meet power calculation estimates for participant sample size while minimizing the number of clinics randomized. We will enroll 18 KPWA primary care clinics and match them by geographic location and size, creating matched clinic pairs, and then randomize the matched clinic pairs to either the intervention or usual care arm, to yield 9 clinics randomized to intervention and 9 to usual care.

Participant Eligibility within Clinic

The study participant sample for each intervention and usual care matched clinic pair will be identified at the time the intervention is implemented at the intervention clinic. For opioid and sedative-hypnotic medications, eligible participants are aged 60 years or older, while for skeletal muscle relaxants, tricyclic antidepressants, and first-generation antihistamines, eligible participants are aged 65 years or older. Eligible participants must be long-term users of the target medication, defined as pharmacy dispensing of at least one of the target medication classes for at least 70 of the prior 90 days. Further, eligible participants must either be assigned to a primary care provider (PCP) or have had 1+ visits in the prior year with a PCP at one of the 18 KPWA clinics participating in the study. Once a participant is determined as having received care or having a PCP at a given clinic, their clinic assignment is fixed, and therefore their randomization assignment will be static throughout the study.

4.2 Exclusion Criteria

Individuals will be excluded for any one of the following reasons, all ascertained pragmatically (i.e., from electronic data sources): a) diagnosis of dementia or a prescription for a medication used to treat dementia (i.e., a cholinesterase inhibitor or memantine); b) residence in a skilled nursing facility; c) metastatic cancer diagnosis in the prior 12 months; d) receiving hospice or palliative care; e) legally blind (unable to read print materials); f) indication the participant requires a translator (cannot read materials printed in English); g) enrolled in other KPWA opioid deprescribing research studies; h) enrolled in a KPWA pharmacy-driven initiative to reduce opioid dose; or i) diagnosed with opioid use disorder.

4.3 Study Enrollment Procedures

This project will request a waiver of consent to send out the educational brochure and collect data from the electronic medical record (EMR), because mailed educational material could reasonably be sent to patients from their healthcare home as part of routine care, and because decisions about any changes to medications will rest with the patient, their care partner, and their PCP. Therefore, there will not be a process for the participant to opt-out of the study. See section 11.2 for details.

1. Before commencing data pulls, the study team will email the Medical Chief at each intervention clinic to alert them that the intervention is going to commence.

2. Using KPWA automated data under a waiver of HIPAA authorization, the study programmer will identify all potential participants who meet inclusion criteria.

5 STUDY INTERVENTIONS

5.1 Interventions, Administration and Duration

The STOP-FALLS intervention consists of two major components: patient education and provider decision support.

Patient Education. Patient education consists of educational brochures and self-care/symptom management handouts. Educational brochures were adapted from prior deprescribing trials conducted in Canada for three of the medication classes targeted by STOP-FALLS: opioids, sedative-hypnotics (benzodiazepines and Z-drugs), and first-generation antihistamines (20, 22). We adapted these materials with input from KPWA delivery system members, including clinical and pharmacy leadership and PCPs. The sedative-hypnotic and antihistamine brochures had been originally designed for older adults. The opioid brochure had been developed for a general population; we extensively revised it to focus on older adults and safety concerns in concordance with KPWA pain management guidelines. The study investigators developed new brochures for skeletal muscle relaxants and tricyclic antidepressants, as no pre-existing materials were available, and modeled them after those from the Canadian deprescribing trials. Patient input was obtained on the opioid, skeletal muscle relaxant, and tricyclic antidepressant brochures through a series of focus groups conducted with KPWA enrollees representative of our target study sample.

Self-care/symptom management handouts were created by the study psychologist (BB) for each of the following symptoms for which a target medication is often prescribed: anxiety, chronic pain, insomnia, and allergies. They emphasize non-pharmacological strategies and describe resources for managing symptoms. Handouts covering the relevant symptom(s) will be mailed along with an educational brochure. In addition, the Centers for Disease Control and Prevention's (CDC) "What YOU Can Do to Prevent Falls" pamphlet will be included with each mailing; the rationale for this pamphlet is to highlight that there are several actions that can be taken to reduce the risk of falls, so that even if a participant does make any changes to their medications, they may take other steps to prevent falls.

Provider Decision Support. Provider decision support consists of two elements: An evidence-based pharmaceutical opinion (EBPO) and "deprescribing pearls". The EBPOs, modeled after

those of the D-PRESCRIBE trial (20), describe the risks associated with the target medication class, alternative evidence-based treatments that could be tried to help a participant reduce their use of the medication, and hyperlinks to practice supports for deprescribing (e.g., pharmacy consultation, mental health referral, and self-care support tools). Prior to intervention implementation, each clinic will receive a 30-minute presentation on the study methods and patient and provider materials, with an emphasis that changes to medication prescriptions are up to their clinical discretion.

In addition to EBPOs, “deprescribing pearls” will be distributed to all intervention clinic PCPs, regardless of whether they have a participant enrolled. Providers in other settings have endorsed the need for guidance on how to initiate deprescribing discussions (23). The content of the pearls was developed by STOP-FALLS investigators based on the published literature (24-26). Each pearl also gives several examples of how to broach discussions of deprescribing with patients, referred to as “conversation starters”. The pearls were modeled after “clinical pearls” used by KPWA to disseminate clinical information updates and thus are anticipated to feel familiar to providers. Table 1 lists the topics for the thirteen pearls.

Table 1: Deprescribing pearl topics sent to primary care providers at all intervention clinics.

	“Deprescribing Pearl” Topics
1	Medicines Linked to Falls
2	Sedative-Hypnotics
3	Opioids
4	Over-the-counter (OTC) Sleep Aids
5	Skeletal Muscle Relaxants
6	Tricyclic Antidepressants
7	Managing Benzodiazepine and Z-Drug Withdrawal Symptoms
8	Fight Prescribing Inertia
9	Pursuing Opportunities for Opioid Deprescribing
10	Deprescribing and the Patient-Provider Relationship
11	Return of Symptoms from Underlying Condition
12	Deprescribing Triggers
13	Deprescribing OTC Antihistamines

All participant and provider materials have been carefully cross-referenced with KPWA clinical practice guidelines and reviewed by leaders in the KPWA delivery system so that all

information and recommendations are concordant with KPWA guidance.

Pilot Testing

Study procedures and intervention materials were pilot-tested within a single intervention clinic (N=142) and matched control clinic (N=160). Participants in the pilot will be excluded from the main trial. The pilot tested study procedures for identifying patients via the EMR, sending mailed materials to participants and faxing decision support to providers. A clinician champion provided feedback to the site principal investigator (BB) on intervention acceptability from the clinic and provider perspective. Key points conveyed included an appreciation for the focus on deprescribing, a minor concern for the intervention generating additional clinic visits as a result of patients receiving intervention materials, and a strong preference for transmission of provider decision support via secure messaging in the EMR rather than fax. The pilot also determined that participants who received an opioid brochure had higher rates of declining to receive further mailings and have their healthcare utilization and pharmacy data used for research purposes. As a result of the latter finding, the study team sought and received IRB approval for a waiver of consent for identifying participants on the premise that differential refusals would bias a pragmatic trial.

Intervention Delivery. For each intervention clinic, materials will be mailed to a subset (approximately one-third) of eligible participants at intervals (mailing “waves”) to minimize burden on the healthcare system that might otherwise result from a large volume of requests from participants for appointments to discuss study materials with their PCP. For each wave, participants who have upcoming visits with their PCP will be prioritized, then participants without an upcoming visit will be selected to ensure that the target sample size for the trial is achieved. If a participant is identified as having a prescription for more than one of the target medication classes, they will be mailed a corresponding brochure at least 90 days after the mailing of the prior brochure. In these cases, brochures will be mailed in the following order: opioids, sedative-hypnotics, skeletal muscle relaxants, tricyclic antidepressants, and/or antihistamines. In light of prior research with KPWA’s older enrollees demonstrating high utilization of over-the-counter, first-generation antihistamines (i.e., non-prescription antihistamines), all participants will receive an antihistamine brochure regardless of whether they have a KPWA pharmacy record of an antihistamine prescription.

Providers will receive a staff message via the EMR, synchronous with a brochure being mailed to a participant, that identifies that participant by name and gives the target medication class of the brochure that they were mailed. The staff message will include a hyperlink to the STOP-FALLS study website where the complete EBPO pertaining to that target medication class can be found.

Each deprescribing pearl will be sent in an e-mail to the clinic chief or other identified “clinical champion” at two-week intervals. The recipient will distribute and promote the information in ways that are appropriate and consistent with how information is typically delivered at that clinic (e.g., emails, weekly meetings, daily huddles, posts, etc.).

<u>Acceptability of Intervention</u>. To evaluate the acceptability of the intervention, 30 days after

each medication brochure mailing date, intervention participants will be mailed a brief postcard questionnaire asking which brochure they received, how useful the information was, and how likely it is that they will have a conversation with their provider about their medication. Response to the questionnaire is voluntary, and data will be collected anonymously. No other direct contact with participants for data collection will occur.

5.2 Handling of Study Interventions

Given this is a cluster randomized trial there is no concealment of the randomization. The randomization occurred once for all clinics participating in the trial.

There is no “training fidelity” plan given the nature of this intervention.

There is no “intervention fidelity” plan given the nature of this intervention.

5.3 Concomitant Interventions

We do not have any concomitant interventions.

5.4 Adherence Assessment

There is no “adherence assessment” plan given the nature of this intervention.

6 STUDY PROCEDURES

The study procedures are outlined in section 5.1, "Interventions, Administration and Duration."

6.1 Schedule of Evaluations

There is no "schedule of evaluations" given the nature of this intervention.

7 SAFETY ASSESSMENTS

7.1 Specifications of Safety Parameters

Comprehensive medication management is part of the KPWA standard of care, and any medication changes will be made by the participant and their PCP. This is consistent with usual care. There is no requirement for participants, care partners or PCPs to engage in discussion about medication safety or deprescribing. The intervention is education and designed to offer participants and their care partners information to understand the risks of CNS-active medications for older adults. The intervention does not itself alter a participant's medication prescriptions.

There is still the possibility for adverse effects from the intervention. These are described under 7.3., below.

7.2 Methods and Timing for Assessing, Recording and Analyzing Safety Parameters

This research involves testing an educational intervention, and therefore the main risk is breach of confidentiality. For more detail on the steps we will take to ensure patient confidentiality, see section 11.3.

To allow for the possibility that adverse events may result from the intervention, we define adverse events and reporting procedures in the following section.

Throughout the study period, any clinic champion or PCP will be able to report safety concerns via the study's project manager's voicemail.

7.3 Adverse Events (AE) and Serious Adverse Events (SAE)

AEs for this study include:

- Medication withdrawal symptoms (e.g., for opioids – nausea, vomiting, diarrhea)

The severity of AE is likely to be moderate and would be *expected* as a result of reduction in medications especially if the medication were withdrawn rapidly or abruptly.

SAEs for this study include:

- Serious adverse drug withdrawal events (ADWE), defined as urgent care, emergency department visit or hospitalization for management of medication withdrawal symptoms for participants mailed an opioid or benzodiazepine brochure
- Unintentional overdose
- Death due to any cause

Deaths would be *expected* given the nature of the population under study (i.e., older adults). Serious ADWE and unintentional overdoses would be *unexpected*, since medication reduction is expected to occur under PCP supervision.

7.3.1 Reporting Procedures

Medically treated falls, safety events, and deaths are trial outcomes and therefore not reportable to the IRB. The one exception is for safety events related to an ADWE. The study team will monitor the six-month period following the last intervention mailing for ADWE through chart review; those events determined to be "definite" will be reported to the IRB.

Contact information (telephone / voicemail) for the study's project manager will be provided to intervention clinics, and clinic champions and PCPs will be invited to report any possible adverse events resulting from the trial. For each report, the site PI and study PI will review the potential concern.

All deaths will be reported to KP Interregional IRB (KPiIRB) within 24 hours of the study team's knowledge of death.

All unanticipated problems (UPs) will be reported to KPiIRB, within 5 business days of the study team's knowledge of the event.

The summaries of all previously reported unexpected and related SAEs, deaths, and UPs, *as well as* all other SAEs and AEs will be reported to KPiIRB at a minimum every 12 months at the time of continuing review.

7.3.2 Follow-up for Adverse Events

Adverse events will be reviewed by KPiIRB for the study as described above, who will determine the need for any follow-up actions and define the exact nature of those actions.

7.4 Safety Monitoring

Other than what is outlined in section 7.3.2, there is no additional safety monitoring plan in place.

8 INTERVENTION DISCONTINUATION

We do not have intervention discontinuation criteria. Participants may request to not receive subsequent mailed intervention materials. Otherwise, participants are considered enrolled and their healthcare utilization and prescription data will be included in analyses.

9 STATISTICAL CONSIDERATIONS

9.1 General Design Issues

Descriptive statistics will be computed and appropriate graphical summaries (e.g., histograms, boxplots, scatterplots) will be generated for all variables across the intervention and control clinics to assess the comparability of baseline characteristics and follow-up times of each group. Although we will match and randomize on size and location of clinics and expect the KPWA population to stay relatively stable over the study time period, any participant characteristics that differ between groups at baseline, and which are known to be related to the outcome or likelihood of disenrollment from the health plan, will be adjusted for in analyses. We will include geographic region of the participant's clinic, age, sex, and an indicator of prior falls in the adjusted models. Statistical significance will be indicated by a P-value <0.05, and all tests and confidence intervals will be two-sided.

9.2 Sample Size and Randomization

Our sample size calculations for the number of clinics to randomize were designed to ensure at least 80% power to detect a 20% reduction in the hazard rate of medically treated falls between the intervention and usual care clinics over 18 months of follow-up. Given the rollout to clinics over time, follow-up is expected to range from 12-26 months. For the sample size calculations, we chose the midpoint, i.e., 18 months of follow-up.

Sample size calculations were informed by estimates obtained from data on a historical cohort of potentially eligible participants at participating KPWA clinics. This cohort consisted of KPWA members meeting the study eligibility criteria as of January 1, 2018 and included follow-up for medically treated falls for 18 months. The 18-month proportion of medically treated falls was estimated to be 29%, and the intraclass correlation (ICC) was estimated to be <<0.001. We used several approaches (34) to calculate the ICC, and all approaches gave close to a zero ICC; to be conservative, we assumed a 0.001 ICC for our sample size calculations.

To obtain an estimate of the number of potentially eligible participants at each clinic, we pulled additional data on a cohort of eligible members as of January 16, 2020. These data were used to calculate an average cluster size of 183 individuals. To account for variable clinic sizes, we used the smaller harmonic mean cluster size of 154 individuals in the calculation of study power (35). Further, for the purpose of calculating power, the outcome was assumed to be binary, i.e., at least one medically treated fall, as opposed to the time to first medically treated fall, which will be used as the outcome in the primary analysis. We expect minimal censoring in our data (5% annually), in which case the minor simplification of estimating the relative risk based on a binary outcome and inflating the sample size by 15% should be similar to estimating the marginal hazard ratio and is conservative (36). Therefore, this sample size calculation closely mimics our approach given the underlying assumptions going into the marginal hazard model with robust standard errors that we will be applying.

Given the estimates above and their accompanying assumptions, it was determined that randomizing nine clinics to each study arm would provide 89% power to detect a 20% reduction (RR=0.80) in the rate of medically treated falls in the intervention group compared to the usual care group. Power calculations were done using PASS 2019 software Version 19.0.1 (37) using a test for two proportions in a cluster-randomized design (38).

9.2.1 Treatment Assignment Procedures

Clinics will be randomized to intervention or control using constrained randomization

(39). Using R software version 3.6.1, the study biostatistician will implement the randomization by considering all possible arrangements of the clinic pairs and with one clinic in each pair assigned to intervention and one to control. Using estimates of the number of eligible members at each clinic, the difference in the average clinic size between the intervention and control groups will be estimated for each possible arrangement, and the 10% with the smallest average difference in cluster size between intervention and control will be retained. The final assignment will then be selected at random from the remaining possibilities.

9.3 Interim analyses and Stopping Rules

No interim analyses are planned. There are no stopping rules.

9.4 Outcomes

9.4.1 Primary outcome

The primary outcome is a participant's first (incident) medically treated fall post baseline, where baseline is defined as the time point after study enrollment at which a first brochure is mailed (or proxy mailed for usual care participants) (see Participant Timeline section below for details). Medically treated falls will be identified from International Classification of Diseases, Tenth Revision (ICD—10) injury (S or T) codes or musculoskeletal diseases (M) code or fall-related cause of injury (W) codes associated with hospitalizations, emergency department visits, urgent care visits, and primary and specialty care visits. We will exclude injury codes with an associated motor vehicle crash code as the cause of injury, provided these codes are recorded within a three-day window of each other. An incident fall is defined using a 3-month washout period after the last pre-baseline fall if any occurred, to ensure that treatment is for an incident fall event.

9.4.2 Secondary outcomes

We will examine three medication outcomes measured at the level of the participant: discontinuation, sustained discontinuation, and dose reduction of any target medication (Figure 2). Exposure to target medications will be obtained from computerized KPWA pharmacy files, which include drug name, dosage form, strength, amount dispensed and number of days' supply. To summarize over a medication class, we will operationalize dosage as an average standardized daily dose (SDD) based on methodology used previously (27-29). We will use morphine equivalents to standardize across opioids. For each target medication, we will define discontinuation at a given timepoint as having no

medications (SDD=0) across 90 days following that specified timepoint. Sustained discontinuation at a given timepoint is defined as having no medications (SDD = 0) across 180 days following the specified timepoint. Dose reduction at a given time point is defined by taking the difference between the SDD in the 90 days prior to first brochure mailing date (average baseline dose) and the 90 days following the specified timepoint (Figure 2). We use the target medication mailing date as the start of follow-up (or proxy mailing date for usual care clinics). For a participant prescribed multiple target medications who will thus receive multiple brochures (e.g., an opioid and benzodiazepine brochure), when examining the second medication discontinuation or reduction, baseline dose is defined at the time of second medication brochure mailing date, not first brochure mailed. (Figure 2). We will further consider the three medication outcomes summarized across all target medication classes. Overall discontinuation and sustained discontinuation are defined as discontinuation or sustained discontinuation of any target medication class, respectively. Overall dose reduction is defined by taking the average of the dose reductions for each target medication class. We consider these overall medication outcomes to be the main secondary outcomes.

Figure 2. Definition of Medication Outcomes at Primary (6 months) and Additional Timepoints (9 and 12 months)

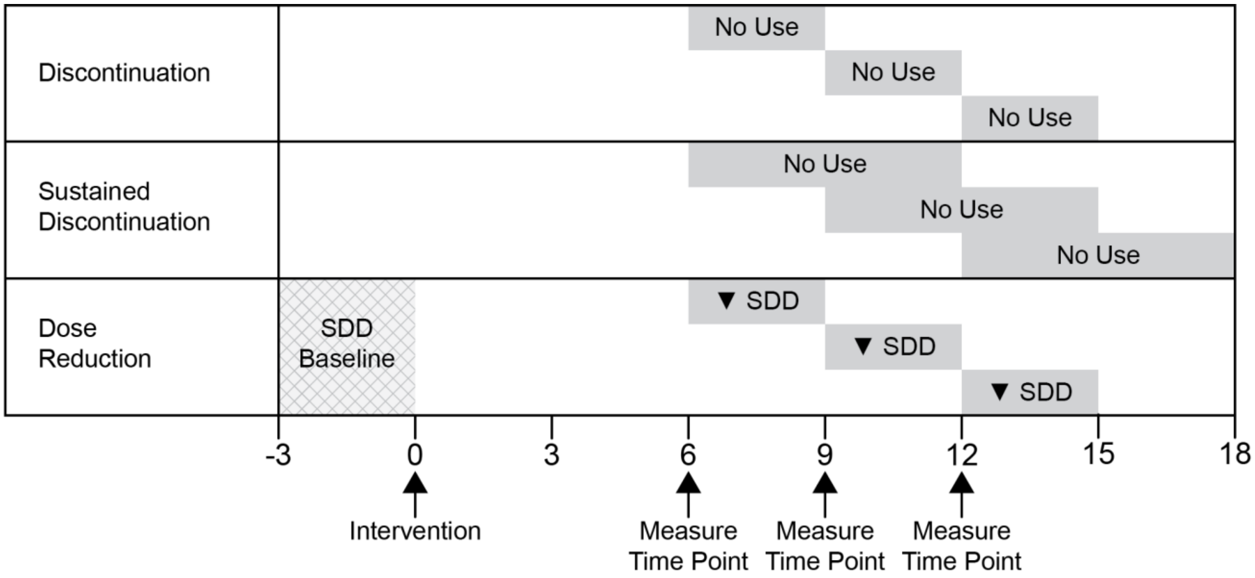

9.5 Data Analyses

Primary Outcome. We will use a time-to-event approach to compare time to first incident medically treated fall between the intervention and usual care groups. This approach accounts for censoring due to disenrollment from the health plan. Since death precludes the observation of a fall, we will use methods that account for competing risks. In this

analysis, we consider non-fall deaths to be a competing risk; deaths that are contemporaneous with a fall event are considered to be medically treated fall outcomes. The statistical literature suggests that using multiple, complementary analysis approaches in competing-risks settings can provide richer information about any effects of an intervention on the outcome of interest and the competing risk (40, 41). We will use robust standard error estimates in all analyses to account for correlation due to cluster randomization and all analyses will include an indicator for intervention and usual care clinic and all adjustment variables.

Follow-up time for all primary outcome analyses is defined relative to the first brochure mailing date for study participants in the intervention group and proxy first brochure mailing date for those in the usual care group. The observed outcome for each participant will include the observation time (the earliest occurrence of first incident medically treated fall, death, disenrollment from the health plan, or the end of the study period), and indicators of whether an incident medically treated fall, death, or disenrollment from the health plan were observed.

Our primary analysis will fit a cause-specific proportional hazards regression model for time to first incident medically treated fall accounting for the competing risk of non-fall death by censoring at the time of non-fall death. We will estimate an adjusted cause-specific hazard ratio for intervention effect and a 95% confidence interval and p-value (42-44). Under this model, we can interpret the hazard ratio as comparing the instantaneous risk of a first incident medically treated fall among those who have not fallen nor died at any given time point between participants in the intervention and usual care groups.

To support this primary analysis, we will fit two further models. The first is a cause-specific proportional hazards regression model for time to non-fall death accounting for the competing risk of incident falls by censoring. Under this model, we can interpret the hazard ratio as comparing the instantaneous risk of death among those who have not fallen nor died at any given time point between participants in the intervention and usual care groups. We will next use a subdistribution hazards model to estimate adjusted cause-specific cumulative incidences of both first medically treated fall and death over time (45, 46). We will display the adjusted cumulative incidence functions graphically. To explore timing of when the intervention may have occurred, we will conduct secondary analyses by comparing the estimated cumulative incidence in the intervention and control arms at 6, 9, and 12 months. We will provide 95% clinic-level bootstrap percentile intervals for the cumulative incidence at these time points.

Finally, we will run a secondary analysis using a composite outcome of time to first incident medically treated fall or death and fit a Cox proportional hazards model (40, 44). We will estimate the hazard ratio and a 95% confidence interval. Under this model, we can interpret the hazard ratio as comparing the instantaneous risk of a first incident medically treated fall or death among those who have neither fallen nor died at any given time point between participants in the intervention and usual care groups. We will also estimate and display graphically Kaplan-Meier estimators of the probability of neither falling nor dying in both

groups, and Nelson-Aalen estimators of the cumulative incidence of falls or death in both groups (43).

Secondary Outcomes. We will examine three medication outcomes measured at the level of the participant: discontinuation, sustained discontinuation, and dose reduction of any target medication and of each target medication (**Figure 2**). The primary time point for all medication analyses is the 6-month time point while 9 and 12 months are secondary time points. To investigate the effectiveness of the intervention on discontinuation and sustained discontinuation (both overall and target-medication-specific), we will fit a Poisson regression model with an indicator of study arm and all other adjustment variables. To investigate the effectiveness of the intervention on dose reduction, we will fit a linear regression model with an indicator of study arm and all other adjustment variables. In these analyses, we will not model the competing risk of death, since we are primarily interested in short-term effectiveness (6 months) and so risk of either death or censoring is small (<5%). We will include follow-up time as an offset term (in the Poisson regression models) or as a weight (in the linear regression model) to account for censoring due to disenrollment from the health plan. To account for clustered data at the clinic level, we will use generalized estimating equations with an independence working correlation structure and a robust sandwich variance estimator and correction for the small number of clinics.

We will also investigate plans to taper medications from the provider and participant perspective. We will capture the providers' perspective from actions documented in the signetur field. We will display summary statistics indicating whether a taper was documented in the signetur field for each target medication over 6 months following the medication-specific brochure mailing date, and the mean time from brochure mailing date to documented taper plan. To capture the participants' perspective on possible tapering, we will use information from the returned postcards. We will present summary statistics indicating the number and proportion of response types tabulated by brochure medication class.

For safety outcomes, we will provide the proportion of participants with a safety outcome by group, given that these are likely to be rare events.

10 DATA COLLECTION AND QUALITY ASSURANCE

10.1 Data Collection Sources

Medically treated falls, medication prescriptions, ADWE, and healthcare utilization will be ascertained via the KPWA EMR and virtual data warehouse. Death and disenrollment are ascertained via the KPWA virtual data warehouse. As previously described, participants are sent a postcard regarding which brochure they received and if they intend to discuss the material with their PCP. These are returned voluntarily and

anonymously and therefore are not linked to the participants' medical record.

10.2 Data Management

KPWA automated data sources and electronic medical records: All automated data will be extracted from KPWA automated data sources. Data will be stored on KPWA password protected computers. Medication deprescribing information will be ascertained by looking at the "sig" field under the "Medication tab" of the KPWA electronic medical record. The results of that chart review, whether or not deprescribing information is indicated and the wording used, will be data-entered into a Microsoft Excel spreadsheet stored on a KPWA password-protected computer. Participant data will be stored on a HIPAA-compliant secure server hosted, managed, and monitored by the Kaiser Permanente Washington Research Institute, with daily backups, and will be deidentified at the earliest possible opportunity. The linking file will be destroyed, per IRB guidelines, 5 years post study end date.

Questionnaires: The responses of the returned questionnaires will be data-entered into a Microsoft Excel spreadsheet stored on a KPWA password protected computer.

11 PARTICIPANT RIGHTS AND CONFIDENTIALITY

11.1 Institutional Review Board (IRB) Review

This protocol and patient/care partner-facing materials will be reviewed and approved by the KPiIRB.

11.2 Informed Consent

<u>Waiver of informed consent.</u> The study is approved by the KPiIRB. Due to the pragmatic and educational nature of the intervention, the KPWA IRB granted a waiver of informed consent for eligible participants consistent with the requirements outlined in 45 CFR 46.116 Part F.3.

<u>Waiver of HIPAA Authorization</u>. A full waiver of HIPAA authorization is approved for study sample identification and collecting study outcomes. The use of protected health information (PHI) for this purpose involves no more than minimal risk to the privacy of individuals, because the PHI will be protected from improper use and disclosure by

virtue of its being accessed only by the KPWA-based study staff. No PHI will be transmitted beyond the KPWA-based programmers and Project Manager, and any linking files created for participant tracking, extraction of data from electronic health records, and data analysis will be destroyed as soon as the research has been completed and the study findings published. The research could not practicably be conducted without the waiver and without access to and use of the PHI, as obtaining consent directly from participants for this purpose would be unnecessarily burdensome.

11.3 Participant Confidentiality

All data will be stored on the KPWA secure network folders accessible only to research study team members. Only the KPWA study programmer will have access to the linking file. The KPWA study programmers, KPWA Project Manager and other study staff will have access to identifiers so that they can recruit participants, mail out study materials, and let providers know that their patient is participating in the study. Computer files will be password-protected with access restricted to staff using this information to perform study-related activities. All analytic data files will be password-protected. Data tables with any identifiers needed for mailing the patient brochures (i.e., name, address)) will be kept separate from all other study data tables. All employees at KPWA routinely sign a confidentiality form that covers access to all data encountered.

11.4 Study Discontinuation

The study may be discontinued at any time by KPiIRB, the CDC, or other government agencies as part of their duties to ensure that research participants are protected.

12 ETHICAL CONSIDERATIONS

All research conducted at KPWA complies with the Department of Health and Human Services requirements for safeguarding the rights and welfare of human subjects, regardless of the source of funding. KPWA and UW each have approved Federal-wide Assurance Compliance filed with the Office for Human Research Protections (OHRP).

13 PUBLICATION OF RESEARCH FINDINGS

Publication of findings from this study will be determined by the study team and may include a protocol paper and a main outcomes paper.

14 REFERENCES

1. Hartholt KA, Stevens JA, Polinder S, van der Cammen TJ, Patka P. Increase in fall-related hospitalizations in the United States, 2001-2008. J Trauma. 2011;71(1):255-8.

2. Gill TM, Desai MM, Gahbauer EA, Holford TR, Williams CS. Restricted activity among community-living older persons: incidence, precipitants, and health care utilization. Ann Intern Med. 2001;135(5):313-21.

3. Tinetti ME, Williams CS. The effect of falls and fall injuries on functioning in community-dwelling older persons. J Gerontol A Biol Sci Med Sci. 1998;53(2):M112-9.

4. Tinetti ME, Williams CS. Falls, injuries due to falls, and the risk of admission to a nursing home. N Engl J Med. 1997;337(18):1279-84.

5. Zijlstra GA, van Haastregt JC, van Eijk JT, van Rossum E, Stalenhoef PA, Kempen GI. Prevalence and correlates of fear of falling, and associated avoidance of activity in the general population of community-living older people. Age Ageing. 2007;36(3):304-9.

6. DeGrauw X, Annest JL, Stevens JA, Xu L, Coronado V. Unintentional injuries treated in hospital emergency departments among persons aged 65 years and older, United States, 2006-2011. J Safety Res. 2016;56:105-9.

7. Hartholt KA, van Beeck EF, Polinder S, van der Velde N, van Lieshout EM, Panneman MJ, et al. Societal consequences of falls in the older population: injuries, healthcare costs, and long-term reduced quality of life. J Trauma. 2011;71(3):748-53.

8. Seppala LJ, Wermelink A, de Vries M, Ploegmakers KJ, van de Glind EMM, Daams JG, et al. Fall-Risk-Increasing Drugs: A Systematic Review and Meta-Analysis: II. Psychotropics. J Am Med Dir Assoc. 2018;19(4):371.e11-.e17.

9. Woolcott JC, Richardson KJ, Wiens MO, Patel B, Marin J, Khan KM, et al. Meta-analysis of the impact of 9 medication classes on falls in elderly persons. Arch Intern Med. 2009;169(21):1952-60.

10. Marcum ZA, Wirtz HS, Pettinger M, LaCroix AZ, Carnahan R, Cauley JA, et al. Anticholinergic medication use and falls in postmenopausal women: findings from the women's health initiative cohort study. BMC Geriatrics. 2016;16(1):76.

11. Gallagher P, Barry P, O'Mahony D. Inappropriate prescribing in the elderly. J Clin Pharm Ther. 2007;32(2):113-21.

12. Summary of the Updated American Geriatrics Society/British Geriatrics Society clinical practice guideline for prevention of falls in older persons. J Am Geriatr Soc. 2011;59(1):148-57.

13. Askari M, Eslami S, van Rijn M, Medlock S, Moll van Charante EP, van der Velde N, et al. Assessment of the quality of fall detection and management in primary care in the Netherlands based on the ACOVE quality indicators. Osteoporos Int. 2016;27(2):569-76.

14. Paniagua MA, Malphurs JE, Phelan EA. Older patients presenting to a county hospital ED after a fall: missed opportunities for prevention. Am J Emerg Med. 2006;24(4):413-7.

15. Bohl AA, Fishman PA, Ciol MA, Williams B, Logerfo J, Phelan EA. A longitudinal analysis of total 3-year healthcare costs for older adults who experience a fall requiring medical care. J Am Geriatr Soc. 2010;58(5):853-60.

16. Laing SS, Silver IF, York S, Phelan EA. Fall prevention knowledge, attitude, and practices of community stakeholders and older adults. J Aging Res. 2011;2011:395357.

17. Anthierens S, Tansens A, Petrovic M, Christiaens T. Qualitative insights into general practitioners views on polypharmacy. BMC Fam Pract. 2010;11:65.

18. Schuling J, Gebben H, Veehof LJ, Haaijer-Ruskamp FM. Deprescribing medication in very elderly patients with multimorbidity: the view of Dutch GPs. A qualitative study. BMC Fam Pract. 2012;13:56.

19. Martin P, Tamblyn R, Ahmed S, Benedetti A, Tannenbaum C. A consumer-targeted, pharmacist-led, educational intervention to reduce inappropriate medication use in community older adults (D-PRESCRIBE trial): study protocol for a cluster randomized controlled trial. Trials. 2015;16:266.

20. Martin P, Tamblyn R, Benedetti A, Ahmed S, Tannenbaum C. Effect of a Pharmacist-Led Educational Intervention on Inappropriate Medication Prescriptions in Older Adults: The D-PRESCRIBE Randomized Clinical Trial. JAMA. 2018;320(18):1889-98.

21. Zwarenstein M, Treweek S, Gagnier JJ, Altman DG, Tunis S, Haynes B, et al. Improving the reporting of pragmatic trials: an extension of the CONSORT statement. BMJ. 2008;337:a2390.

22. Tannenbaum C, Martin P, Tamblyn R, Benedetti A, Ahmed S. Reduction of Inappropriate Benzodiazepine Prescriptions Among Older Adults Through Direct Patient Education: The EMPOWER Cluster Randomized Trial. JAMA Internal Medicine. 2014;174(6):890-8.

23. Bayliss EA, Shetterly SM, Drace ML, Norton J, Green AR, Reeve E, et al. The OPTIMIZE patient- and family-centered, primary care-based deprescribing intervention for older adults with dementia or mild cognitive impairment and multiple chronic conditions: study protocol for a pragmatic cluster randomized controlled trial. Trials. 2020;21(1):542.

24. Henry SG, Gosdin MM, White AEC, Kravitz RL. “It Sometimes Doesn’t Even Work”: Patient Opioid Assessments as Clues to Therapeutic Flexibility in Primary Care. Journal of General Internal Medicine. 2020;35(6):1635-40.

25. Pottie K, Thompson W, Davies S, Grenier J, Sadowski CA, Welch V, et al. Deprescribing benzodiazepine receptor agonists: Evidence-based clinical practice guideline. Can Fam Physician. 2018;64(5):339-51.

26. Reeve E, Moriarty F, Nahas R, Turner JP, Kouladjian O'Donnell L, Hilmer SN. A narrative review of the safety concerns of deprescribing in older adults and strategies to mitigate potential harms. Expert Opin Drug Saf. 2018;17(1):39-49.

27. Gray SL, Anderson ML, Dublin S, Hanlon JT, Hubbard R, Walker R, et al. Cumulative use of strong anticholinergics and incident dementia: a prospective cohort study. JAMA Intern Med. 2015;175(3):401-7.

28. Hanlon JT, Zhao X, Naples JG, Aspinall SL, Perera S, Nace DA, et al. Central Nervous System Medication Burden and Serious Falls in Older Nursing Home Residents. J Am Geriatr Soc. 2017;65(6):1183-9.

29. Von Korff M, Saunders K, Thomas Ray G, Boudreau D, Campbell C, Merrill J, et al. De facto long-term opioid therapy for noncancer pain. Clin J Pain. 2008;24(6):521-7.

30. Hanlon JT, Gray SL. Deprescribing trials: A focus on adverse drug withdrawal events. J Am Geriatr Soc. 2022;70(9):2738-41.

31. Holly Hedegaard MFG. A Revised ICD–10–CM Surveillance Case Definition for Injury-related Emergency Department Visits 2021 [Available from: https://www.cdc.gov/nchs/data/nhsr/nhsr164.pdf.

32. Vivolo-Kantor A, Pasalic E, Liu S, Martinez PD, Gladden RM. Defining indicators for drug overdose emergency department visits and hospitalisations in ICD-10-CM coded discharge data. Inj Prev. 2021;27(S1):i56-i61.

33. M DS, Boudreau D, Ichikawa L, Cronkite D, Albertson-Junkans L, Salgado G, et al. Primary Care Opioid Taper Plans Are Associated with Sustained Opioid Dose Reduction. J Gen Intern Med. 2020;35(3):687-95.

34. Chakraborty H, Hossain A. R package to estimate intracluster correlation coefficient with confidence interval for binary data. Comput Methods Programs Biomed. 2018;155:85-92.

35. Moulton RJHLH. Cluster Randomised Trials. 1st ed. New York: Chapman and Hall/CRC; 2009. 338 p.

36. Shan G. Two-stage optimal designs based on exact variance for a single-arm trial with survival endpoints. J Biopharm Stat. 2020;30(5):797-805.

37. PASS 2019 Power Analysis and Sample Size Software NCSS, LLC. Kaysville, Utah, USA, ; 2019 [Available from: ncss.com/software/pass.

38. Flynn TN. Design and Analysis of Cluster Randomization Trials in Health Research.: Allan Donner and Neil Klar. London: Arnold, 2000, pp.178, £35.00. ISBN: 0-340-69153-0. International Journal of Epidemiology. 2001;30(2):407-8.

39. Moulton LH. Covariate-based constrained randomization of group-randomized trials. Clin Trials. 2004;1(3):297-305.

40. Andersen PK, Geskus RB, de Witte T, Putter H. Competing risks in epidemiology: possibilities and pitfalls. Int J Epidemiol. 2012;41(3):861-70.

41. Austin PC, Fine JP. Accounting for competing risks in randomized controlled trials: a review and recommendations for improvement. Stat Med. 2017;36(8):1203-9.

42. Prentice RL, Kalbfleisch JD, Peterson AV, Jr., Flournoy N, Farewell VT, Breslow NE. The analysis of failure times in the presence of competing risks. Biometrics. 1978;34(4):541-54.

43. Lin DY. Cox regression analysis of multivariate failure time data: the marginal approach. Stat Med. 1994;13(21):2233-47.

44. Therneau TM LT, Atkinson E, and Crowson C. survival: survival analysis. R package version 3.4-0 2022 [Available from: https://CRAN.R-project.org/package=survival.

45. Zhou B, Fine J, Latouche A, Labopin M. Competing risks regression for clustered data. Biostatistics. 2012;13(3):371-83.

46. Zhou B, Latouche A. crrSC: competing risks regression for stratified and clustered data. R package version 1.1.2 2022 [Available from: https://CRAN.R-project.org/package=crrSC.

47. Wiltsey Stirman S, Baumann AA, Miller CJ. The FRAME: an expanded framework for reporting adaptations and modifications to evidence-based interventions. Implementation Science. 2019;14(1):58.

48. Proctor EK, Powell BJ, McMillen JC. Implementation strategies: recommendations for specifying and reporting. Implementation Science. 2013;8(1):139.

49. Aarons GA, Ehrhart MG, Farahnak LR. The implementation leadership scale (ILS): development of a brief measure of unit level implementation leadership. Implementation Science. 2014;9(1):45.

50. Hansen PG. The Definition of Nudge and Libertarian Paternalism: Does the Hand Fit the Glove? European Journal of Risk Regulation. 2016;7(1):155-74.

51. Vlaev I, King D, Dolan P, Darzi A. The Theory and Practice of “Nudging”: Changing Health Behaviors. Public Administration Review. 2016;76.

52. Donohue JM, Huskamp HA, Wilson IB, Weissman J. Whom do older adults trust most to provide information about prescription drugs? Am J Geriatr Pharmacother. 2009;7(2):105-16.

53. Chaudhuri S, Le T, White C, Thompson H, Demiris G. Examining health information-seeking behaviors of older adults. Comput Inform Nurs. 2013;31(11):547-53.

54. Sak G, Schulz PJ. Exploring Health Information-Seeking Preferences of Older Adults With Hypertension: Quasi-Experimental Design. JMIR Cardio. 2018;2(1):e12.
