# Supplementary material for: Reducing Central Nervous System–Active Medications to Prevent Falls and Injuries Among Older Adults: A Cluster Randomized Clinical Trial
Source: JAMA Netw Open. 2024 Jul 25;7(7):e2424234. doi: 10.1001/jamanetworkopen.2024.24234 (PMC11273227; doi:10.1001/jamanetworkopen.2024.24234)
Supplement: Supplement 2. — eTable 1. Adverse Drug Withdrawal Event Codes eTable 2. Unintentional Overdose Codes eTable 3. Heterogeneity of Treatment Effect for Time to First Medically Treated Fall and Non-Fall Death eTable 4. Summary of Medication Outcomes After 9 Months From Mailing for a Given Medication eTable 5. Summary of Medication Outcomes After 12 Months From Mailing for a Given Medication eTable 6. Summary of Medication Outcomes After 15 Months From Mailing for a Given Medication eAppendix. Evidence-Based Pharmaceutical Opinions [file jamanetwopen-e2424234-s002.pdf]

Supplementary Online Content

Phelan EA, Williamson BD, Balderson BH, et al. Reducing central nervous system–active medications to prevent falls and injuries among older adults: a cluster randomized clinical trial. *JAMA Netw Open.* 2024;7(7):e2424234. doi:10.1001/jamanetworkopen.2024.24234

eTable 1. Adverse Drug Withdrawal Event Codes

eTable 2. Unintentional Overdose Codes

eTable 3. Heterogeneity of Treatment Effect for Time to First Medically Treated Fall and Non-Fall Death

eTable 4. Summary of Medication Outcomes After 9 Months From Mailing for a Given Medication

eTable 5. Summary of Medication Outcomes After 12 Months From Mailing for a Given Medication

eTable 6. Summary of Medication Outcomes After 15 Months From Mailing for a Given Medication

eAppendix. Evidence-Based Pharmaceutical Opinions

This supplementary material has been provided by the authors to give readers additional information about their work.

© 2024 Phelan EA et al. *JAMA Network Open.*

eTable 1. Adverse Drug Withdrawal Event Codes

ICD	Code	Definition
		**Opioids**
10	T40.2X5	Adverse effect of other opioids
10	F11.12	Opioid abuse with withdrawal
10	F11.23	Opioid dependence, with withdrawal
10	F11.13	Opioid abuse with withdrawal
10	F11.93	Opioid use, unspecified with withdrawal
10	R10.9	Unspecified abdominal pain
10	R10.83	Colic
10	R10.84	Generalized abdominal pain
10	F43.0	Acute stress reaction
10	F43.9	Reaction to severe stress, unspecified
10	F43.89	Other reactions to severe stress
10	R45.0	Nervousness
10	R45.1	Restlessness and agitation
10	R45.7	State of emotional shock and stress, unspecified
10	R45.4	Irritability and anger
10	R45.5	Hostility
10	R45.6	Violent behavior
10	R19.7	Diarrhea, unspecified
10	R42	Dizziness and giddiness
10	R45.1	Restlessness and agitation
10	R45.0	Nervousness
10	K30	Functional dyspepsia
10	K52.9	Noninfective gastroenteritis and colitis, unspecified
10	G47.00	Insomnia, unspecified
10	G47.9	Sleep disorder, unspecified
10	R50.2	drug induced fever
10	R50.9	Fever, unspecified
10	R03.0	Elevated blood-pressure reading, without diagnosis of hypertension
10	R25.2	Cramp and spasm
10	M79.10	Myalgia, unspecified site
10	M79.11	Myalgia of mastication muscle
10	M79.12	Myalgia of auxiliary muscles, head and neck
10	M79.18	Myalgia, other site
10	H57.04	Mydriasis
10	R11.0	Nausea
10	R11.1	Vomiting
10	R11.10	Vomiting, unspecified
10	R11.11	Vomiting without nausea
10	R11.2	Nausea with vomiting, unspecified
10	L98.9	Disorder of the skin and subcutaneous tissue, unspecified
10	G25.81	Restless legs syndrome
10	J34.8	Other specified disorders of nose and nasal sinuses

© 2024 Phelan EA et al. *JAMA Network Open.*

ICD	Code	Definition
10	R56.9	Unspecified convulsions
10	R06.7	Sneezing
10	R61	Generalized hyperhidrosis
10	R00.0	Tachycardia, unspecified
10	R20.8	Other disturbances of skin sensation
10	R20.9	Unspecified disturbances of skin sensation
10	R20.3	Hyperesthesia
10	R25.1	Tremor, unspecified
10	R25.9	Unspecified abnormal involuntary movements
10	G25.1	Drug-induced tremor
10	G25.70	Drug induced movement disorder, unspecified
10	G25.79	Other drug induced movement disorders
10	R06.89	Other abnormalities of breathing
10	R06.9	Unspecified abnormalities of breathing
10	M25.50	Pain in unspecified joint
10	R09.89	Runny nose
10	H04.20	Unspecified epiphora
**Benzodiazepines**		
10	T42.4X5	Adverse effect of benzodiazepines
10	F41.8	Other specified anxiety disorders
10	F41.9	Anxiety disorder, unspecified
10	F13.980	Sedative, hypnotic, or anxiolytic-induced
10	F11.988	Induced, without use disorder anxiety disorder
10	G47.00	Insomnia, unspecified
10	G47.9	Sleep disorder, unspecified
10	F51.5	Nightmare disorder
10	R25.1	Tremor, unspecified
10	R25.9	Unspecified abnormal involuntary movements
10	G25.1	Drug-induced tremor
10	G25.70	Drug induced movement disorder, unspecified
10	G25.79	Other drug induced movement disorders
10	R61	Generalized hyperhidrosis
10	R11.0	Nausea
10	R11.1	Vomiting
10	R11.10	Vomiting, unspecified
10	R11.11	Vomiting without nausea
10	R11.2	Nausea with vomiting, unspecified
10	R00.2	Palpitations
10	R00.0	Tachycardia, unspecified
10	R51.9	Headache, unspecified
10	G44.4	Drug-induced headache, not elsewhere classified
10	G44.40	Drug-induced headache, not elsewhere classified, not intractable
10	G44.41	Drug-induced headache, not elsewhere classified, intractable

© 2024 Phelan EA et al. *JAMA Network Open.*

10	R56.9	Unspecified convulsions
10	R45.4	Irritability and anger
10	R45.5	Hostility
10	R45.6	Violent behavior
10	F43.0	Acute stress reaction
10	F43.9	Reaction to severe stress, unspecified
10	F43.89	Other reactions to severe stress
10	R45.0	Nervousness
10	R45.1	Restlessness and agitation
10	R45.7	State of emotional shock and stress, unspecified
10	R44.0	Auditory hallucinations
10	R44.1	Visual hallucinations
10	R44.2	Other hallucinations
10	R44.3	Hallucinations, unspecified
10	R44.8	Other symptoms and signs involving general sensations and perceptions
10	R44.9	Unspecified symptoms and signs involving general sensations and perceptions
10	F41.0	Panic disorder [episodic paroxysmal anxiety]
10	R41.0	Disorientation, unspecified
10	F13.13	Sedative, hypnotic or anxiolytic abuse with withdrawal
10	F13.130	Sedative, hypnotic or anxiolytic abuse with withdrawal, uncomplicated
10	F13.131	Sedative, hypnotic or anxiolytic abuse with withdrawal delirium
10	F13.132	Sedative, hypnotic or anxiolytic abuse with withdrawal with perceptual disturbance
10	F13.139	Sedative, hypnotic or anxiolytic abuse with withdrawal, unspecified
10	F13.23	Sedative, hypnotic or anxiolytic dependence with withdrawal
10	F13.230	Sedative, hypnotic or anxiolytic dependence with withdrawal, uncomplicated
10	F13.231	Sedative, hypnotic or anxiolytic dependence with withdrawal delirium
10	F13.232	Sedative, hypnotic or anxiolytic dependence with withdrawal with perceptual disturbance
10	F13.239	Sedative, hypnotic or anxiolytic dependence with withdrawal, unspecified
10	F13.93	Sedative, hypnotic or anxiolytic use, unspecified with withdrawal
10	F13.930	Sedative, hypnotic or anxiolytic use, unspecified with withdrawal, uncomplicated
10	F13.931	Sedative, hypnotic or anxiolytic use, unspecified with withdrawal delirium
10	F13.932	Sedative, hypnotic or anxiolytic use, unspecified with withdrawal with perceptual disturbances
10	F13.939	Sedative, hypnotic or anxiolytic use, unspecified with withdrawal, unspecified
10	F32.A	Depression, unspecified (acute)
10	R42	Dizziness and giddiness
10	R50.2	drug-induced fever

© 2024 Phelan EA et al. *JAMA Network Open.*

10	R50.9	Fever, unspecified
10	R53.8	Other malaise and fatigue
10	R53.81	Other malaise
10	R53.83	Other fatigue
10	G90.8	Other disorders of autonomic nervous system
10	G90.9	Disorder of the autonomic nervous system, unspecified
10	H53.10	Unspecified subjective visual disturbances
10	H53.14	Visual discomfort
10	H53.15	Visual distortions of shape and size
10	H53.16	Psychophysical visual disturbances
10	H53.19	Other subjective visual disturbances
10	H53.8	Other visual disturbances
10	H53.9	Unspecified visual disturbance
10	H53.71	Glare sensitivity
10	H81.9	Unspecified disorder of vestibular function
10	H81.4	Vertigo of central origin
10	R61	Generalized hyperhidrosis
10	F23	paranoid (acute)
10	M79.10	Myalgia, unspecified site
10	M79.11	Myalgia of mastication muscle
10	M79.12	Myalgia of auxiliary muscles, head and neck
10	M79.18	Myalgia, other site
10	R29.2	Abnormal reflex
10	R20.2	Paresthesia of skin
10	R20.0	Anesthesia of skin
10	R20.1	Hypoesthesia of skin
10	R20.8	Other disturbances of skin sensation
10	R20.9	Unspecified disturbances of skin sensation
10	R20.3	Hyperesthesia
10	R27.0	Ataxia, unspecified
10	R27.8	Other lack of coordination
10	R27.9	Unspecified lack of coordination

© 2024 Phelan EA et al. *JAMA Network Open.*

eTable 2. Unintentional Overdose Codes

ICD	Code	Definition
10	T40.2X1	Poisoning by other opioids, accidental (unintentional)
10	T40.2X1A	Poisoning by other opioids, accidental (unintentional), initial encounter
10	T40.2X1D	Poisoning by other opioids, accidental (unintentional), subsequent encounter
10	T40.2X1S	Poisoning by other opioids, accidental (unintentional), sequela
10	T40.3X1	Poisoning by methadone, accidental (unintentional)
10	T40.3X1A	Poisoning by methadone, accidental (unintentional), initial encounter
10	T40.3X1D	Poisoning by methadone, accidental (unintentional), subsequent encounter
10	T40.3X1S	Poisoning by methadone, accidental (unintentional), sequela
10	T40.411	Poisoning by fentanyl or fentanyl analogs, accidental (unintentional)
10	T40.411A	Poisoning by fentanyl or fentanyl analogs, accidental (unintentional), initial encounter
10	T40.411D	Poisoning by fentanyl or fentanyl analogs, accidental (unintentional), subsequent encounter
10	T40.411S	Poisoning by fentanyl or fentanyl analogs, accidental (unintentional), sequela
10	T40.421	Poisoning by tramadol, accidental (unintentional)
10	T40.421A	Poisoning by tramadol, accidental (unintentional), initial encounter
10	T40.421D	Poisoning by tramadol, accidental (unintentional), subsequent encounter
10	T40.421S	Poisoning by tramadol, accidental (unintentional), sequela
10	T40.491	Poisoning by other synthetic narcotics, accidental (unintentional)
10	T40.491A	Poisoning by other synthetic narcotics, accidental (unintentional), initial encounter
10	T40.491D	Poisoning by other synthetic narcotics, accidental (unintentional), subsequent encounter
10	T40.491S	Poisoning by other synthetic narcotics, accidental (unintentional), sequela
10	T40.601	Poisoning by unspecified narcotics, accidental (unintentional)
10	T40.601A	Poisoning by unspecified narcotics, accidental (unintentional), initial encounter
10	T40.601D	Poisoning by unspecified narcotics, accidental (unintentional), subsequent encounter
10	T40.601S	Poisoning by unspecified narcotics, accidental (unintentional), sequela
10	T40.691	Poisoning by other narcotics, accidental (unintentional)
10	T40.691A	Poisoning by other narcotics, accidental (unintentional), initial encounter

© 2024 Phelan EA et al. *JAMA Network Open.*

ICD	Code	Definition
10	T40.691D	Poisoning by other narcotics, accidental (unintentional), subsequent encounter
10	T40.691S	Poisoning by other narcotics, accidental (unintentional), sequela
10	T42.4X1	Poisoning by benzodiazepines, accidental (unintentional)
10	T42.4X1A	Poisoning by benzodiazepines, accidental (unintentional), initial encounter
10	T42.4X1D	Poisoning by benzodiazepines, accidental (unintentional), subsequent encounter
10	T42.4X1S	Poisoning by benzodiazepines, accidental (unintentional), sequela
10	T42.6X1	Poisoning by other antiepileptic and sedative-hypnotic drugs, accidental (unintentional)
10	T42.6X1A	Poisoning by other antiepileptic and sedative-hypnotic drugs, accidental (unintentional), initial encounter
10	T42.6X1D	Poisoning by other antiepileptic and sedative-hypnotic drugs, accidental (unintentional), subsequent encounter
10	T42.6X1S	Poisoning by other antiepileptic and sedative-hypnotic drugs, accidental (unintentional), sequela
10	T43.011	Poisoning by tricyclic antidepressants, accidental (unintentional)
10	T43.011A	Poisoning by tricyclic antidepressants, accidental (unintentional), initial encounter
10	T43.011D	Poisoning by tricyclic antidepressants, accidental (unintentional), subsequent encounter
10	T43.011S	Poisoning by tricyclic antidepressants, accidental (unintentional), sequela
10	T45.0X1	Poisoning by antiallergic and antiemetic drugs, accidental (unintentional)
10	T45.0X1A	Poisoning by antiallergic and antiemetic drugs, accidental (unintentional), initial encounter
10	T45.0X1D	Poisoning by antiallergic and antiemetic drugs, accidental (unintentional), subsequent encounter
10	T45.0X1S	Poisoning by antiallergic and antiemetic drugs, accidental (unintentional), sequela
10	T48.1X1	Poisoning by skeletal muscle relaxants [neuromuscular blocking agents], accidental (unintentional)
10	T48.1X1A	Poisoning by skeletal muscle relaxants [neuromuscular blocking agents], accidental (unintentional), initial encounter
10	T48.1X1D	Poisoning by skeletal muscle relaxants [neuromuscular blocking agents], accidental (unintentional), subsequent encounter
10	T48.1X1S	Poisoning by skeletal muscle relaxants [neuromuscular blocking agents], accidental (unintentional), sequela

© 2024 Phelan EA et al. *JAMA Network Open.*

eTable 3. Heterogeneity of Treatment Effect[a] for Time to First Medically Treated Fall and Non-Fall Death

	**Cause-specific outcome analysis: time to first medically-treated fall**		**Cause-specific outcome analysis: time to non-fall death**	
**Variable**	**Ratio of HRs (95% CI)**	**p-value from LRT**	**Ratio of HRs (95% CI)**	**p-value from LRT**
Age (< 80 vs 80+)	0.996 (0.732, 1.356)	0.98	0.737 (0.226, 2.405)	0.59
Sex assigned at birth	0.964 (0.727, 1.278)	0.79	1.903 (0.767, 4.725)	0.21
Any fall prior to baseline	1.008 (0.778, 1.305)	0.95	1.014 (0.331, 3.109)	0.98
Multimorbidity	1.016 (0.855, 1.207)	0.91	0.87 (0.114, 6.642)	0.83
Frailty	1.107 (0.826, 1.484)	0.71	--[b]	--

Abbreviations: HR, hazard ratio; LRT, likelihood ratio test; CI, confidence interval

[a]All analyses used a cause-specific Cox proportional hazards model for a given outcome censoring for disenrollment, study follow-up end, or for the competing risk outcome if applicable (e.g. for time to medically treated fall the analysis would censor for death). All models adjusted for geographic region of the clinic, age, sex assigned at birth, and any falls prior to baseline. A non-fall death is defined as death with no preceding fall care.

[b]The regression model with an interaction between frailty and intervention arm failed to converge, because no deaths were observed among those with frailty in the usual care arm (and only one death was observed among those with frailty in the intervention arm).

© 2024 Phelan EA et al. *JAMA Network Open.*

eTable 4. Summary of Medication Outcomes After 9 Months From Mailing for a Given Medication

	Analysis Population	Usual Care (N=1261)	Intervention (N=1106)		
**BINARY OUTCOME**	N	Adj Rate (95 % CI)[a]	Adj Rate (95 % CI)	Adj RR (95% CI)	P-Value
**Discontinuation (90 days SDD=0 post 9 months)**					
Opioid	1588	0.06 (0.05, 0.08)	0.06 (0.04, 0.07)	0.89 (0.63, 1.25)	0.498
Benzodiazepine/Z-drug	465	0.29 (0.25, 0.33)	0.34 (0.31, 0.39)	1.20 (1.01, 1.42)	0.039
Tricyclic antidepressant	273	0.16 (0.12, 0.21)	0.30 (0.24, 0.38)	1.95 (1.36, 2.8)	0.000
Muscle relaxant	184	0.42 (0.33, 0.52)	0.41 (0.31, 0.54)	0.98 (0.68, 1.43)	0.933
Antihistamine (Rx)	55	0.03 (0.00, 0.19)	0.25 (0.12, 0.54)	9.60 (1.17, 79.09)	0.036
First Target Med[b]	2288	0.12 (0.10, 0.14)	0.15 (0.13, 0.18)	1.27 (1.00, 1.62)	0.049
**Sustained Discontinuation (180 days SDD=0 post 9 months)**					
Opioid	1588	0.06 (0.04, 0.08)	0.05 (0.04, 0.06)	0.88 (0.60, 1.29)	0.512
Benzodiazepine/Z-drug	465	0.28 (0.23, 0.33)	0.32 (0.28, 0.37)	1.15 (0.93, 1.41)	0.190
Tricyclic antidepressant	273	0.11 (0.07, 0.15)	0.24 (0.18, 0.32)	2.23 (1.39, 3.57)	0.001
Muscle relaxant	184	0.38 (0.28, 0.52)	0.37 (0.30, 0.46)	0.97 (0.66, 1.43)	0.864
Antihistamine (Rx)	55	0.03 (0.00, 0.18)	0.24 (0.10, 0.54)	8.95 (1.03, 77.38)	0.047
First Target Med[b]	2288	0.11 (0.09, 0.13)	0.14 (0.12, 0.15)	1.25 (1.00, 1.57)	0.047
**CONTINUOUS OUTCOME**	N	Adj Mean (95 % CI)[d]	Adj Mean (95 % CI)	Adj Diff (95% CI)	P-Value
**Dose Reduction[c]**					
Opioid	1588	-0.31 (-0.42, -0.20)	-0.34 (-0.47, -0.21)	-0.03 (-0.20, 0.13)	0.694
Benzodiazepine/Z-drug	465	-1.22 (-1.43, -1.00)	-1.23 (-1.42, -1.03)	-0.01 (-0.30, 0.29)	0.953
Tricyclic antidepressant	273	-1.15 (-1.60, -0.70)	-1.89 (-2.45, -1.34)	-0.74 (-1.40, -0.08)	0.029
Muscle relaxant	184	-2.90 (-3.31, -2.48)	-2.95 (-3.39, -2.51)	-0.06 (-0.67, 0.56)	0.859
Antihistamine (Rx)	55	-0.13 (-0.24, -0.02)	-0.18 (-0.39, 0.03)	-0.04 (-0.30, 0.21)	0.730
First Target Med	2288	-0.58 (-0.75, -0.41)	-0.72 (-0.82, -0.62)	-0.14 (-0.33, 0.05)	0.138

© 2024 Phelan EA et al. *JAMA Network Open.*

Abbreviations: Adj, adjusted; RR, relative risk; CI, confidence interval; SDD, standardized daily dose; Rx, prescription; med, medication; diff, difference

[a]Adjusted rates, relative risks, and corresponding 95% confidence and p-values are calculated from a Poisson regression model for the given binary outcome with an offset for proportion of days enrolled within outcome window at 9 months (90 days for discontinuation and 180 days for sustained discontinuation). Model fit using generalized estimating equations to account for correlation due to clinic randomization using small number of cluster correction. All analyses adjusted for baseline SDD dose of the given medication analyzed, age, sex, geographic region, and baseline prior fall. Adjusted rates are calculated at the population mean level of the covariate.

[b]First target medication is the first medication mailed to the participant at the time of study enrollment.

[c]Dose Reduction is defined as the change in medication dose at 9 months (average SDD over 90 days post 9 months) minus baseline dose (average SDD over 90 days pre-baseline).

[d]Adjusted means, mean differences and corresponding 95% confidence intervals and p-values are calculated from a weighted regression model for the continuous outcome dose reduction with the weight being number of days enrolled in the 90 day outcome window at 9 months fit using generalized estimating equations to account for correlation due to clinic randomization using a small number of cluster correction. All analyses adjusted for baseline SDD dose of the given medication analyzed, age, sex, geographic region, and baseline prior fall. Adjusted means are calculated at the population mean level of the covariate.

© 2024 Phelan EA et al. *JAMA Network Open.*

eTable 5. Summary of Medication Outcomes After 12 Months From Mailing for a Given Medication

BINARY OUTCOME	Analysis Population N	Usual Care (N=1261) Adj Rate (95 % CI)[a]	Intervention (N=1106) Adj Rate (95 % CI)	Adj RR (95% CI)	P-Value
**Discontinuation (90 days SDD=0 post 12 months)**					
Opioid	1588	0.07 (0.05, 0.11)	0.08 (0.06, 0.10)	1.01 (0.66, 1.55)	0.954
Benzodiazepine/Z-drug	465	0.43 (0.40, 0.46)	0.44 (0.39, 0.50)	1.02 (0.88, 1.17)	0.806
Tricyclic antidepressant	273	0.21 (0.16, 0.27)	0.40 (0.32, 0.50)	1.93 (1.39, 2.68)	0.000
Muscle relaxant	184	0.44 (0.34, 0.58)	0.42 (0.34, 0.51)	0.95 (0.67, 1.33)	0.749
Antihistamine (Rx)	55	0.09 (0.04, 0.20)	0.29 (0.14, 0.59)	3.24 (1.18, 8.86)	0.022
First Target Med**	2288	0.15 (0.14, 0.17)	0.18 (0.15, 0.22)	1.19 (0.96, 1.48)	0.113
**Sustained Discontinuation (180 days SDD=0 post 12 months)**					
Opioid	1588	0.07 (0.05, 0.11)	0.06 (0.04, 0.09)	0.88 (0.55, 1.40)	0.589
Benzodiazepine/Z-drug	465	0.43 (0.39, 0.47)	0.42 (0.35, 0.50)	0.97 (0.80, 1.18)	0.791
Tricyclic antidepressant	273	0.20 (0.16, 0.26)	0.39 (0.31, 0.49)	1.95 (1.4, 2.73)	0.000
Muscle relaxant	184	0.40 (0.27, 0.61)	0.45 (0.37, 0.54)	1.11 (0.69, 1.79)	0.664
Antihistamine (Rx)	55	0.09 (0.04, 0.19)	0.24 (0.12, 0.51)	2.81 (1.1, 7.19)	0.031
First Target Med[b]	2288	0.15 (0.13, 0.17)	0.17 (0.14, 0.21)	1.14 (0.88, 1.47)	0.310

CONTINUOUS OUTCOME	N	Adj Mean (95 % CI)[d]	Adj Mean (95 % CI)	Adj Diff (95% CI)	P-Value
**Dose Reduction[c]**					
Opioid	1588	-0.34 (-0.47, -0.20)	-0.34 (-0.46, -0.22)	0.00 (-0.19, 0.18)	0.980
Benzodiazepine/Z-drug	465	-1.29 (-1.51, -1.08)	-1.23 (-1.42, -1.04)	0.07 (-0.24, 0.37)	0.662
Tricyclic antidepressant	273	-1.67 (-1.96, -1.38)	-2.63 (-3.17, -2.09)	-0.96 (-1.60, -0.32)	0.003
Muscle relaxant	184	-2.99 (-3.45, -2.52)	-3.11 (-3.48, -2.74)	-0.13 (-0.74, 0.49)	0.687
Antihistamine (Rx)	55	-0.22 (-0.35, -0.09)	-0.30 (-0.53, -0.07)	-0.08 (-0.35, 0.20)	0.586
First Target Med	2288	-0.66 (-0.79, -0.53)	-0.74 (-0.85, -0.62)	-0.07 (-0.25, 0.10)	0.423

© 2024 Phelan EA et al. *JAMA Network Open.*

Abbreviations: Adj, adjusted; RR, relative risk; CI, confidence interval; SDD, standardized daily dose; Rx, prescription; med, medication; diff, difference

[a]Adjusted rates, relative risks, and corresponding 95% confidence and p-values are calculated from a Poisson regression model for the given binary outcome with an offset for proportion of days enrolled within outcome window at 12 months (90 days for discontinuation and 180 days for sustained discontinuation). Model fit using generalized estimating equations to account for correlation due to clinic randomization using small number of cluster correction. All analyses will adjust for baseline SDD dose of the given medication analyzed, age, sex, geographic region, and baseline prior fall. Adjusted rates are calculated at the population mean level of the covariate.

[b]First target medication is the first medication mailed to the participant at the time of study enrollment.

[c]Dose Reduction is defined as the change in medication dose at 6 months (average SDD over 90 days post 12 months) minus baseline dose (average SDD over 90 days pre-baseline).

[d]Adjusted means, mean differences and corresponding 95% confidence intervals and p-values are calculated from a weighted regression model for the continuous outcome dose reduction with the weight being number of days enrolled in the 90 day outcome window at 12 months fit using generalized estimating equations to account for correlation due to clinic randomization using a small number of cluster correction. All analyses adjusted for baseline SDD dose of the given medication analyzed, age, sex, geographic region, and baseline prior fall. Adjusted means are calculated at the population mean level of the covariate.

© 2024 Phelan EA et al. *JAMA Network Open.*

eTable 6. Summary of Medication Outcomes After 15 Months From Mailing for a Given Medication

BINARY OUTCOME	Analysis Population N	Usual Care (N=1261) Adj Rate (95 % CI)[a]	Intervention (N=1106) Adj Rate (95 % CI)	Adj RR (95% CI)	P-Value
**Discontinuation (90 days SDD=0 post 15 months)**					
Opioid	1588	0.09 (0.07, 0.11)	0.10 (0.08, 0.12)	1.06 (0.79, 1.41)	0.718
Benzodiazepine/Z-drug	465	0.46 (0.42, 0.50)	0.43 (0.37, 0.51)	0.95 (0.79, 1.14)	0.564
Tricyclic antidepressant	273	0.33 (0.26, 0.42)	0.54 (0.46, 0.63)	1.61 (1.25, 2.08)	0.000
Muscle relaxant	184	0.49 (0.36, 0.68)	0.55 (0.45, 0.66)	1.10 (0.75, 1.62)	0.612
Antihistamine (Rx)	55	0.11 (0.04, 0.30)	0.26 (0.14, 0.48)	2.34 (0.85, 6.49)	0.101
First Target Med[b]	2288	0.18 (0.17, 0.20)	0.21 (0.18, 0.24)	1.12 (0.93, 1.35)	0.219

CONTINUOUS OUTCOME	N	Adj Mean (95 % CI)[d]	Adj Mean (95 % CI)	Adj Diff (95% CI)	P-Value
**Dose Reduction[c]**					
Opioid	1588	-0.35 (-0.50, -0.21)	-0.41 (-0.54, -0.27)	-0.06 (-0.26, 0.15)	0.586
Benzodiazepine/Z-drug	465	-1.45 (-1.70, -1.20)	-1.22 (-1.47, -0.97)	0.23 (-0.14, 0.61)	0.227
Tricyclic antidepressant	273	-1.66 (-1.95, -1.37)	-2.69 (-3.26, -2.12)	-1.03 (-1.71, -0.36)	0.003
Muscle relaxant	184	-3.04 (-3.53, -2.56)	-3.27 (-3.72, -2.81)	-0.22 (-0.94, 0.49)	0.537
Antihistamine (Rx)	55	-0.23 (-0.35, -0.10)	-0.3 (-0.54, -0.07)	-0.08 (-0.36, 0.20)	0.582
First Target Med	2288	-0.64 (-0.77, -0.51)	-0.73 (-0.84, -0.61)	-0.09 (-0.26, 0.09)	0.337

© 2024 Phelan EA et al. *JAMA Network Open.*

Abbreviations: Adj, adjusted; RR, relative risk; CI, confidence interval; SDD, standardized daily dose; Rx, prescription; med, medication; diff, difference

[a]Adjusted rates, relative risks, and corresponding 95% confidence and p-values are calculated from a Poisson regression model for the given binary outcome with an offset for proportion of days enrolled within outcome window at 15 months (90 days for discontinuation and 180 days for sustained discontinuation). Model fit using generalized estimating equations to account for correlation due to clinic randomization using small number of cluster correction. All analyses will adjust for baseline SDD dose of the given medication analyzed, age, sex, geographic region, and baseline prior fall. Adjusted rates are calculated at the population mean level of the covariate.

[b]First target medication is the first medication mailed to the participant at the time of study enrollment.

[c]Dose Reduction is defined as the change in medication dose at 15 months (average SDD over 90 days post 15 months) minus baseline dose (average SDD over 90 days pre-baseline).

[d]Adjusted means, mean differences and corresponding 95% confidence intervals and p-values are calculated from a weighted regression model for the continuous outcome dose reduction with the weight being number of days enrolled in the 90 day outcome window at 15 months fit using generalized estimating equations to account for correlation due to clinic randomization using a small number of cluster correction. All analyses adjusted for baseline SDD dose of the given medication analyzed, age, sex, geographic region, and baseline prior fall. Adjusted means are calculated at the population mean level of the covariate.

© 2024 Phelan EA et al. *JAMA Network Open.*

Evidence-based Pharmaceutical Opinion: First-Generation Antihistamines

The 2019 American Geriatrics Society Beers List[1] of potentially inappropriate medications recommends avoiding use of first-generation antihistamines in adults aged 65+. High-quality evidence indicates these medications increase risk of cognitive impairment, delirium, dementia[2] and urinary retention.

[1] American Geriatrics Society 2019 Updated AGS Beers Criteria® for Potentially Inappropriate Medication Use in Older Adults. American Geriatrics Society Beers Criteria® Update Expert Panel. *J Am Geriatr Soc* 2019;67:674-694.

[2] Gray SL, Anderson ML, Dublin S et al. Cumulative use of strong anticholinergics and incident dementia: A prospective cohort study. *JAMA Intern Med* 2015;175:401-407. Available at: https://www.ncbi.nlm.nih.gov/pmc/articles/PMC4358759/

Suggested Strategies

Taper Medication*

- **Route to pharmacy pool for consult {.HRMCONSULT}** to obtain a tapering schedule. You as the provider will need to initiate the taper and work with the patient.

*Although most patients do not require a taper of antihistamines, patients using higher doses for anxiety may benefit from a taper to minimize discontinuation symptoms. For patients who suffer discontinuation symptoms despite a gradual taper, the duration of the taper can be extended depending upon the pace that the patient can tolerate.

☐ **Psychotherapy**. For psychotherapy for anxiety or insomnia management, **refer to a Licensed Clinical Social Worker (LICSW) in your clinic** if available **or to KPWA Mental Health and Wellness** Services. The patient may also be referred to the **myStrength self-care app**, free to all KPWA members, for help with anxiety or insomnia. Use the **AVS smart phrase .mystrengthinformation**.

Alternatives for Allergic Rhinitis

- Intranasal saline
- Intranasal corticosteroid (e.g., fluticasone)
- Second generation (oral) antihistamine (e.g., cetirizine, fexofenadine, loratadine)

Alternatives for Anxiety

- Selective serotonin reuptake inhibitor (SSRI) (e.g., sertraline, escitalopram)*
- Selective noradrenaline reuptake inhibitor (SNRI) (e.g., duloxetine)*
- Buspirone

*These medications are preferred over antihistamines because of a more favorable side effect profile. However they may still increase fall risk.

After Visit Summary
.avsanxiety

Alternatives for Insomnia

- Melatonin
- Ramelteon

After Visit Summary
.avsinsomniaptinfo

© 2024 Phelan EA et al. *JAMA Network Open.*

Symptom Monitoring

Brief, validated tools are available in Epic (flowsheets) for tracking changes in symptoms over time and can facilitate medication tapering/dose reduction. In addition to monitoring symptoms of the condition for which this medication was prescribed, consider also monitoring related symptoms. Available tools include:

- PEG Pain Screening Tool (PEG)
- Generalized Anxiety Disorder 7-item scale (GAD-7)
- Patient Health Questionnaire-9 (PHQ-9)
- Insomnia Severity Index (ISI)

KPWA Guidance

See KPWA's Safer Alternatives to Potentially High Risk Medications in the Elderly for more details: http://incontext.ghc.org/rx/mum/documents/hrmchart.pdf

See KPWA's Insomnia Guidelines: http://incontext.ghc.org/clinical/clin_topics/insomnia/insomnia_kpwa.html

STOP-FALLS Research Study
This study has been reviewed and endorsed by KPWA Pharmacy and Primary Care.

UNIVERSITY of WASHINGTON

KAISER PERMANENTE®

Canadian Deprescribing Network

Adapted with permission of Cara Tannenbaum and Institut universitaire de gériatrie de Montréal

© 2024 Phelan EA et al. *JAMA Network Open.*

Evidence-based Pharmaceutical Opinion: Sedative-Hypnotics (Benzodiazepines and Z-drugs)

The 2019 American Geriatrics Society Beers List[1] of drugs to avoid in older adults considers benzodiazepines and Z-drugs as potentially inappropriate medications for adults aged 65+ due to an increased risk of cognitive impairment, falls, fractures, and motor vehicle crashes, even with intermittent use.

[1] American Geriatrics Society 2019 Updated AGS Beers Criteria® for Potentially Inappropriate Medication Use in Older Adults. American Geriatrics Society Beers Criteria® Update Expert Panel. *J Am Geriatr Soc* 2019;67:674-694.

Suggested Strategies

☐ **Taper Medication**

- **Route to pharmacy pool for consult {.HRMCONSULT}** to obtain a tapering schedule. You as the provider will need to initiate the taper and work with the patient.
- **Implement and follow the benzodiazepine tapering schedule as per KPWA guidelines** (see last page for pictorial representation for patients).
- **Implement the Z-drug tapering schedule as per KPWA guidelines:** Decrease the number of days per week that the patient takes the medication (e.g., 6 nights per week x2 weeks, then 5 nights per week x2 weeks, etc).

☐ **Psychotherapy**. For psychotherapy for anxiety or insomnia, **refer to a Licensed Clinical Social Worker (LICSW) in your clinic** if available **or to KPWA Mental Health and Wellness** Services. The patient may also be referred to the **myStrength self-care app**, free to all KPWA members, for help with anxiety or insomnia. Use the **AVS smart phrase .mystrengthinformation**.

☐ **For insomnia**, recommend a **Cognitive Behavioral Therapy workbook**; several are listed in the **After Visit Summary material .avsinsomniaptinfo**.

Symptom Monitoring During Tapering

Brief, validated tools are available in Epic (flowsheets) for tracking changes in symptoms over time and can facilitate medication tapering/dose reduction. In addition to monitoring symptoms of the condition for which this medication was prescribed, consider also monitoring related symptoms. Available tools include:

- PEG Pain Screening Tool (PEG)
- Generalized Anxiety Disorder 7-item scale (GAD-7)
- Patient Health Questionnaire-9 (PHQ-9)
- Insomnia Severity Index (ISI)

© 2024 Phelan EA et al. *JAMA Network Open.*

Alternatives for Anxiety

- Selective serotonin reuptake inhibitor (SSRI) (e.g., sertraline, escitalopram)*
- Selective serotonin norepinephrine reuptake inhibitor (SNRI) (e.g., duloxetine)*
- Buspirone

*These medications are preferred over sedative-hypnotics because of a more favorable side effect profile. However, they may still increase fall risk.

After Visit Summary
.avsanxiety

Alternatives for Insomnia

- Melatonin
- Ramelteon

After Visit Summary
.avsinsomniaptinfo

KPWA Guidance

Safer Alternatives to Potentially High Risk Medications in the Elderly:
http://incontext.ghc.org/rx/mum/documents/hrmchart.pdf

Sedative-hypnotic Guidelines: https://cl.kp.org/wa/cpg/clinical-guidelines/bzd/benzo.html

Insomnia Guidelines: http://incontext.ghc.org/clinical/clin_topics/insomnia/insomnia_kpwa.html

© 2024 Phelan EA et al. *JAMA Network Open.*

An example of how to taper a benzodiazepine

TABLET SIZE / EXPLANATIONS

Full dose · Half dose · Quarter of a dose · No dose

WEEK	MO	TU	WED	TH	FRI	SA	SU	✓
1	Full dose	Full dose	Full dose	Full dose	Full dose	Full dose	Full dose	
2	Half dose	Full dose	Full dose	Half dose	Full dose	Full dose	Full dose	
3	Half dose	Full dose	Full dose	Half dose	Full dose	Full dose	Half dose	
4	Half dose	Full dose	Half dose	Half dose	Full dose	Full dose	Half dose	
5	Half dose	Full dose	Half dose	Half dose	Full dose	Half dose	Half dose	
6	Half dose	Half dose	Half dose	Half dose	Full dose	Half dose	Half dose	
7	Half dose	Half dose	Half dose	Half dose	Half dose	Half dose	Half dose	
8	Quarter of a dose	Half dose	Half dose	Quarter of a dose	Half dose	Half dose	Half dose	
9	Quarter of a dose	Half dose	Quarter of a dose	Quarter of a dose	Half dose	Half dose	Quarter of a dose	
10	Quarter of a dose	Half dose	Quarter of a dose	Quarter of a dose	Half dose	Quarter of a dose	Quarter of a dose	
11	Quarter of a dose	Quarter of a dose	Quarter of a dose	Quarter of a dose	Half dose	Quarter of a dose	Quarter of a dose	
12	Quarter of a dose	Quarter of a dose	Quarter of a dose	Quarter of a dose	Half dose	Quarter of a dose	Quarter of a dose	
13	No dose	Quarter of a dose	Quarter of a dose	No dose	Quarter of a dose	Quarter of a dose	Quarter of a dose	
14	No dose	Quarter of a dose	No dose	No dose	Quarter of a dose	No dose	No dose	
15	No dose	No dose	No dose	No dose	No dose	No dose	No dose	

STOP-FALLS Research Study
This study has been reviewed and endorsed by KPWA Pharmacy and Primary Care.

UNIVERSITY of WASHINGTON · KAISER PERMANENTE · Canadian Deprescribing Network

Adapted with permission of Cara Tannenbaum and Institut universitaire de gériatrie de Montréal

© 2024 Phelan EA et al. *JAMA Network Open.*

Evidence-Based Pharmaceutical Opinion: Opioids for Chronic Non-Cancer Pain

The 2019 American Geriatrics Society Beers List[1] of drugs to avoid in older adults considers opioids as potentially inappropriate medications for adults aged 65+ due to a greater risk of falls, fractures, and unintentional overdose.

[1] American Geriatrics Society 2019 Updated AGS Beers Criteria® for Potentially Inappropriate Medication Use in Older Adults. American Geriatrics Society Beers Criteria® Update Expert Panel. *J Am Geriatr Soc* 2019;67:674-694.

Suggested Strategies

<u>Consider one or more of the following:</u>

☐ **Taper opioid**

- ☐ Generate a tapering schedule that can be given to the patient using the opioid reduction calculator available at **deprescribingnetwork.ca** (under Professionals, Useful Tools). An example tapering schedule produced by this calculator:

Canadian Deprescribing Network | Réseau canadien pour la déprescription

Schedule for reducing the dose of my opioid medication

Oxycodone (OxyNEO)

My current dose is : **20 mg**

I take my opioid : **twice a day**

I want to reduce my dose every : **1 week(s)**

I want to start reducing my dose on (DD-MM-YYYY) : **01-07-2020**

Week Beginning	Take in the morning	Take at night	Your capsules may look like this
01-07-2020	15 mg	15 mg	15
08-07-2020	10 mg	10 mg	10
15-07-2020	0	10 mg	10
22-07-2020	Completed	Completed	Completed

- ☐ E-Consult to CHRONIC PAIN CONSULTATION for opioid taper schedule recommendations.
- ☐ Refer to Clinical Pharmacist Management Team (COMET). COMET provides longitudinal opioid taper management including a taper plan, withdrawal symptom management, opioid risk mitigation, patient education, and optimization of non-opioid analgesics and adjuvants. If you and your patient have decided to taper and agree to be managed by COMET then you may put in a referral by entering an order for “REF Clinical Pharmacist Managed Opioid Taper Program.”

☐ For a CBT psychotherapy approach to chronic pain management, recommend the patient use the myStrength self-care app, free to all KPWA members. Use the AVS smart phrase **.mystrengthinformation**.

Condition	Evidence-based recommendations	Quality of evidence	Strength of recommendation*
**Chronic Low Back Pain**	Exercise<br>Acupuncture<br>Mindfulness-based stress reduction	*Moderate*	**Strong**
	Yoga<br>Tai chi<br>Progressive relaxation<br>Cognitive behavioral therapy<br>Spinal manipulation (chiropractic care)	*Low*	**Strong**
**Chronic Neck Pain**	Thoracic and cervical manipulation or mobilization (chiropractic care or osteopathic physician)<br>Physical therapy	*Moderate*	**Moderate**
	Neck, shoulder, girdle, and trunk exercises	*Low*	**Weak**
**Chronic Knee and/or Hip Pain**	Weight loss (if overweight)	*Very low*	**Strong**
	Land-based exercise (hip and knee strengthening exercises, walking, tai chi)	*Low (knee); Moderate (hip)*	**Strong**
	Heat packs<br>Corticosteroid injection<br>Offloading joint (cane or walker)<br>Aquatic exercise<br>Duloxetine	*Very low*<br>*Very low*<br>*Low (knee); Very low (hip)*<br>*Low*<br>*Moderate (knee); Low (hip)*	**Conditional For†**
	Topical non-steroidal anti-inflammatory drugs (NSAIDs)	*Moderate*	**Conditional Neutral‡**
	Cushioned supportive shoes/avoid high heels<br>Topical capsaicin cream (for hip)	*Very low*	**Conditional Neutral‡**
	Cognitive behavioral therapy for pain coping or psychological symptoms	*Low (for knee); Very low (for hip)*	**Conditional Neutral‡**

* Strength of recommendation ratings incorporate a variety of considerations, including expert opinion and context and thus do not always align with quality of evidence ratings.

† "Conditional For" denotes treatments that could be offered although there is uncertainty over the balance of benefits; consider personal preferences in decision-making.

‡ "Conditional Neutral" indicates that the guideline working group could not determine the direction of the recommendation.

REFERENCES

Qaseem et al. Noninvasive treatments for acute, subacute, and chronic low back pain: A clinical practice guideline from the American College of Physicians. Ann Intern Med. 2017 Apr 4;166(7):514-530.

KPWA Back Pain Guideline. Available at: http://incontext.ghc.org/clinical/clin_topics/backpain/backpain_kpwa.html

Neck Pain: Revision 2017 clinical practice guidelines linked to the International Classification of Functioning Disability and Health from the American Physical Therapy Association. J Orthop Sports Phys Ther. 2017;47(7):A1-A83. Available at: https://www.jospt.org/doi/pdf/10.2519/jospt.2017.0302

Royal Australian College of General Practitioners (RACGP): Guideline for the management of knee and hip osteoarthritis, 2nd edition, 2018. Available at: https://www.racgp.org.au/download/Documents/Guidelines/Musculoskeletal/guideline-for-the-management-of-knee-and-hip-oa-2nd-edition.pdf

KPWA Chronic Opioid Therapy Guideline. Available at: https://cl.kp.org/wa/cpg/clinical-guidelines/cot/cot.html

© 2024 Phelan EA et al. *JAMA Network Open.*

Adapted with permission of Cara Tannenbaum and Institut universitaire de gériatrie de Montréal

© 2024 Phelan EA et al. *JAMA Network Open.*

Evidence-based Pharmaceutical Opinion: Skeletal Muscle Relaxants

The 2019 American Geriatrics Society Beers List[1] of drugs to avoid in older adults considers skeletal muscle relaxants as potentially inappropriate medications for adults aged 65+ due to a greater risk of fractures. Because they have anticholinergic side effects, these agents may also increase dementia risk.[2]

[1] American Geriatrics Society 2019 Updated AGS Beers Criteria® for Potentially Inappropriate Medication Use in Older Adults. American Geriatrics Society Beers Criteria® Update Expert Panel. *J Am Geriatr Soc;* 00:1–21, 2019.

[2] Gray SL, Anderson ML, Dublin S et al. Cumulative use of strong anticholinergics and incident dementia: A prospective cohort study. *JAMA Intern Med* 2015;175:401-407. Available at: https://www.ncbi.nlm.nih.gov/pmc/articles/PMC4358759/

Suggested Strategies

☐ **Taper Medication**

- **Route to pharmacy pool for consult {.HRMCONSULT}** to obtain a tapering schedule. You as the provider will need to initiate the taper and work with the patient.

☐ **Add a pharmacologic alternative**, as applicable depending on the type of pain:

- Scheduled acetaminophen
- Topical analgesics (diclofenac, lidocaine, capsaicin)
- Localized injections (e.g., corticosteroids)
- Gabapentin (for neuropathic pain)*
- Duloxetine (for osteoarthritis-related low back or knee pain)†

*Gabapentin may still increase fall risk. Use lowest effective dose.

† Duloxetine is associated with falls but may have long-term efficacy for osteoarthritis.

See KPWA's Safer Alternatives to Potentially High Risk Medications in the Elderly for more details: http://incontext.ghc.org/rx/mum/documents/hrmchart.pdf

☐ For a **CBT psychotherapy approach** to chronic pain management, recommend the patient use the **myStrength** self-care app, free to all KPWA members. Use the AVS smart phrase **.mystrengthinformation**.

☐ **Recommend/refer for non-pharmacological intervention(s)** for persistent musculoskeletal pain:

- Physical therapy
- Mind-body practices (tai chi, yoga)
- Relaxation techniques
- Acupuncture
- Massage
- Psychotherapy for pain management (cognitive behavioral therapy [CBT]–pain, mindfulness-based stress reduction [MBSR], acceptance and commitment therapy [ACT]–pain)
- Assistive device use (cane or walker) to offload weight from affected joint(s)
- Surgical treatment for severe persistent pain (e.g., joint replacement, spinal surgery)

© 2024 Phelan EA et al. *JAMA Network Open.*

After Visit Summary

An After Visit Summary template is available for persistent pain: **.avspainchronic**.

© 2024 Phelan EA et al. *JAMA Network Open.*

Evidence-based Pharmaceutical Opinion: Tricyclic Antidepressants

To minimize discontinuation symptoms, progressively taper the dose of the medication for at least two to four weeks. For patients who suffer discontinuation symptoms despite a gradual taper, the duration of the taper can be extended beyond four weeks, depending on the pace that the patient can tolerate.

Symptom Monitoring

Brief, validated tools are available in Epic (flowsheets) for tracking changes in symptoms over time and can facilitate medication tapering/dose reduction. In addition to monitoring pain, consider monitoring anxiety, depression, and sleep. These tools include:

- PEG Pain Screening Tool (PEG)
- Generalized Anxiety Disorder 7-item scale (GAD-7)
- Patient Health Questionnaire-9 (PHQ-9)
- Insomnia Severity Index (ISI)

© 2024 Phelan EA et al. *JAMA Network Open.*

Tapering Recommendations

The 2019 American Geriatrics Society Beers List[1] of potentially inappropriate medications recommends avoiding use of tricyclic antidepressants in older adults. High-quality evidence indicates these medications increase risk of falls, fractures, cognitive impairment, dementia,[2] and urinary retention.

[1] American Geriatrics Society 2019 Updated AGS Beers Criteria® for Potentially Inappropriate Medication Use in Older Adults. American Geriatrics Society Beers Criteria® Update Expert Panel. *J Am Geriatr Soc;* 00:1–21, 2019.

[2] Gray SL, Anderson ML, Dublin S et al. Cumulative use of strong anticholinergics and incident dementia: A prospective cohort study. *JAMA Intern Med* 2015;175:401-407. Available at: https://www.ncbi.nlm.nih.gov/pmc/articles/PMC4358759/

Suggested Strategies

☐ **Taper Medication**

Route to pharmacy pool for consult if needed {.HRMCONSULT} to obtain a tapering schedule. You as the provider will need to initiate the taper and work with the patient.

To minimize discontinuation symptoms, progressively taper the dose of the medication by a fixed amount for at least two to four weeks. For patients who suffer discontinuation symptoms despite a gradual taper, the taper duration can be extended beyond four weeks, depending on the pace that the patient can tolerate.

☐ **Psychotherapy**. For psychotherapy for depression and/or insomnia management, **refer to a Licensed Clinical Social Worker (LICSW) in your clinic** if available **or to KPWA Mental Health and Wellness** Services. The patient may also be referred to the **myStrength self-care app**, free to all KPWA members, for help with depression, insomnia, or chronic pain. Use the **AVS smart phrase .mystrengthinformation**.

Alternatives for Depression

- Selective serotonin reuptake inhibitors (SSRIs) (e.g., sertraline, escitalopram)*
- Selective serotonin norepinephrine reuptake inhibitors (SNRIs) (e.g., duloxetine, venlafaxine)*

*These medications are preferred over TCAs because of a more favorable side effect profile. However they may still increase fall risk.

After Visit Summary
.avsdepressionwithmeds or
.avsdepressionwithoutmeds

Alternatives for Insomnia

- Melatonin
- Ramelteon

After Visit Summary
.avsinsomniaptinfo

Alternatives for Chronic Pain

- Topical agents (e.g., capsaicin, lidocaine)
- Gabapentin*
- Duloxetine*

*These medications are preferred over TCAs because of their lower risk profile. However they may still increase fall risk.

Non-pharmacological Interventions for Chronic Pain

- Psychotherapy for pain management (cognitive behavioral therapy [CBT]–pain, mindfulness-based stress reduction [MBSR], acceptance and commitment therapy [ACT]–pain)
- Assistive device use (cane or walker) to offload weight from affected joint(s)
- Surgical treatment for severe persistent pain (e.g., joint replacement, spinal surgery)
- Physical therapy
- Mind-body practices (tai chi, yoga)
- Relaxation techniques
- Acupuncture
- Massage

After Visit Summary
.avspainchronic

© 2024 Phelan EA et al. *JAMA Network Open.*

Deprescribing Pearls
TIPS FOR DAILY PRACTICE

Brief, validated tools are available in Epic (flowsheets) for tracking changes in symptoms over time and can facilitate medication tapering/dose reduction. In addition to monitoring symptoms of the condition for which this medication was prescribed, consider also monitoring related symptoms. Available tools include:

- PEG Pain Screening Tool (PEG)
- Generalized Anxiety Disorder 7-item scale (GAD-7)
- Patient Health Questionnaire-9 (PHQ-9)
- Insomnia Severity Index (ISI)

KPWA Guidance

See KPWA's Safer Alternatives to Potentially High Risk Medications in the Elderly for more details: http://incontext.ghc.org/rx/mum/documents/hrmchart.pdf

STOP-FALLS Research Study
This study has been reviewed and endorsed by KPWA Pharmacy and Primary Care.

© 2024 Phelan EA et al. *JAMA Network Open.*

Symptom Monitoring during Tapering

© 2024 Phelan EA et al. *JAMA Network Open.*

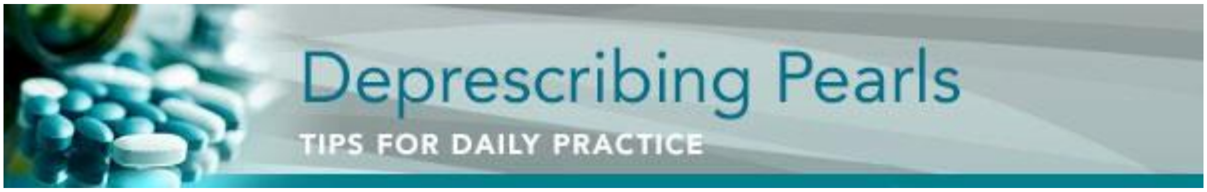

Medicines Linked to Falls*

Many older adults are not aware medicines increase fall risk:

- Less than one-third of older adults taking a medicine linked to falls know that the medicine increases the risk of falls.
- However, approximately 60% of older adults are willing to reduce or stop their medicine if their physician recommends it.

How to start the conversation with your patient:

- “You may have received a brochure at home regarding your medicines and the risk of falls. Would it be okay if we talked about that today?”
- “As people age, their bodies handle medicines differently. A medicine that was safe for you in the past may not be safe anymore. I’d like to talk with you about adjusting your medications so that your regimen is as safe for you as possible. How does that sound?”
- “I am worried about your use of [medication]. This medication has been linked to falls. I have some ideas about how we might work together on adjusting your medications to lower your fall risk. Would you like to talk about it?”

**Medication classes linked with falls in older adults include: opioids, benzodiazepines, z-drugs, skeletal muscle relaxants, tricyclic antidepressants, and first-generation antihistamines.*

Resources:

- For more information about tips and evidence-based tools for deprescribing, click here for short (3 minute) videos.
- For a printable PDF pocket guide to the 2019 American Geriatrics Society Beers Criteria, click here.

STOP-FALLS Research Study

Email: Monica.M.Fujii@kp.org
Phone: 1-888-324-3166

© 2024 Phelan EA et al. *JAMA Network Open.*

KAISER PERMANENTE

UNIVERSITY *of* WASHINGTON

© 2024 Phelan EA et al. *JAMA Network Open.*

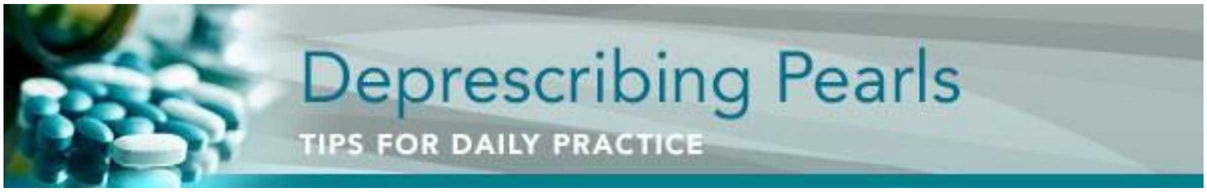

Sedative-Hypnotics

Safer treatments than sedative-hypnotics exist for treating anxiety and insomnia in older adults:

- Benzodiazepines and z-drugs only increase sleep by a small amount (zolpidem: 11 minutes), and tolerance may develop quickly. Risks often outweigh benefits, including unawareness of activities during sleeping (e.g., driving) and next day impairment.
- Benzodiazepines and z-drugs increase the risk of falls and fractures, even when used for short periods of time or as needed.
- Cognitive Behavioral Therapy for Insomnia (CBTi) has been found to improve sleep onset, total amount of sleep, and sleep quality and is safer than benzodiazepines and z-drugs.
- CBTi and a written tapering protocol are tools that can help patients successfully stop their sedative-hypnotics.
- You can easily refer patients to effective resources using dot phrases:
    - .avsinsomniaptinfo (AVS, information on sleep hygiene)
    - .mystrengthinformation (a referral to the MyStrength app including interactive modules based on CBTi principles)

How to start the conversation with your patient:

- “I’m worried about you staying on your sleeping pill. Although it may be effective for a short time, it is not the best long-term treatment. Would you be willing to talk about other options?”
- “Even if you take a low dose or have not had problems with this medicine in the past, your body changes over time, and you become more vulnerable to side effects as you get older. How does trying a lower dose of [medication] sound?”
- “Many people have slowly reduced this medicine and found other ways to help manage their sleep problems and anxiety. We can talk about what you can try instead of [medication]. Are you willing to try to get off [medication] if we work together on it?”

Resources:

© 2024 Phelan EA et al. *JAMA Network Open.*

- For more information about tips and evidence-based tools for deprescribing, click here for short (3 minute) videos.
- KPWA clinical guidelines for:
    - Benzodiazepine and Z-Drug Safety here.
    - Insomnia here.

STOP-FALLS Research Study

Email: Monica.M.Fujii@kp.org
Phone: 1-888-324-3166

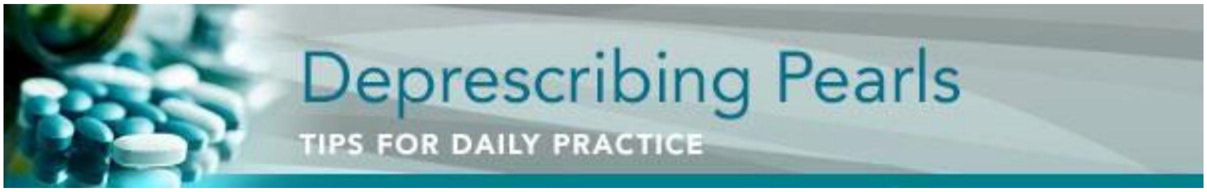

Opioids

Many older adults manage pain without opioids and avoid opioid-associated risks:

- After discontinuing opioids, some patients report positive outcomes such as less pain, improved function, and better quality of life.
- Opioids can cause drowsiness, dizziness, or confusion, which can lead to car accidents, falls, and even death.
- The rate of opioid-related hospitalizations is increasing fastest among adults aged 65 and older.
- Opioids can lead to dependence and addiction even after taking them for a short time.
- Evidence supports the use of oral (acetaminophen) and topical treatments (NSAIDs, lidocaine, heat) and self-management strategies such as psychotherapy for the management of chronic non-cancer pain in older adults.

How to start the conversation with your patient:

- "I am concerned about your continued use of opioids. Over time, opioids often stop helping with pain. However, they can continue to cause serious side effects. It may not sound possible but many people with pain actually feel better after they stop their opioid. Have you ever heard of that?
- "Many people have slowly reduced their opioid dose and found safer treatments to manage their pain and improve their everyday function. Would you be willing to talk about it?"
- "I am concerned about your opioid use. There are a lot of risks and it doesn't seem to be making your pain and functioning to where either of us would like it to be. Would it be okay for us to discuss this more today?"
- "I know pain is a major concern for you, and you've been on opioids a long time. I would like to review how this is going and explore safer, long-term alternatives. How does that sound to you?"

Resources:

- For more information about tips and evidence-based tools for deprescribing, click here for short (3 minute) videos.

© 2024 Phelan EA et al. *JAMA Network Open.*

- KPWA clinical guidelines and practice resources:
    - Chronic Opioid Therapy (COT) Safety for Patients on COT for Chronic Non-malignant Pain here.
    - Non-specific Back Pain here.
    - Pain Management Consulting Teams here.
    - For an opioid tapering AVS that includes patient self-care strategies, use .OPIOIDTAPERLETTER

STOP-FALLS Research Study

Email: Monica.M.Fujii@kp.org
Phone: 1-888-324-3166

KAISER PERMANENTE

UNIVERSITY of WASHINGTON

Deprescribing Pearls
TIPS FOR DAILY PRACTICE

Over-the-counter (OTC) Sleep Aids

Many older adults take OTC antihistamines for sleep:

- More than 20% of older adults report using OTC sleep aids. However, the majority do not discuss OTC sleep aids with their healthcare provider.
- Older adults are often unaware of OTC sleep aid side effects.
- Some may be taking more than one product containing an OTC antihistamine, without realizing it, thus getting a high dose.
- OTC antihistamines can cause confusion and increase the risk of falls, and long-term use may increase dementia risk.

How to start the conversation with your patient:

- “Do you take anything for sleep that you buy from the pharmacy, grocery store, or online without a prescription? I am asking because some of these medications can be harmful to your thinking and can increase your risk of falling.”
- “These medications are sold by many different names, most commonly Benadryl and anything that includes ‘PM’ in the name, for example, Tylenol PM. Have you heard of these?”
- “I’m worried about you staying on this over-the-counter sleep aid. Many people have stopped this medication and found other ways to help manage their sleep problems. Would you be willing to talk about it?”

Resources:

- KPWA clinical guideline for Insomnia here.

STOP-FALLS Research Study

Email: Monica.M.Fujii@kp.org
Phone: 1-888-324-3166

KAISER PERMANENTE® UNIVERSITY *of* WASHINGTON

© 2024 Phelan EA et al. *JAMA Network Open.*

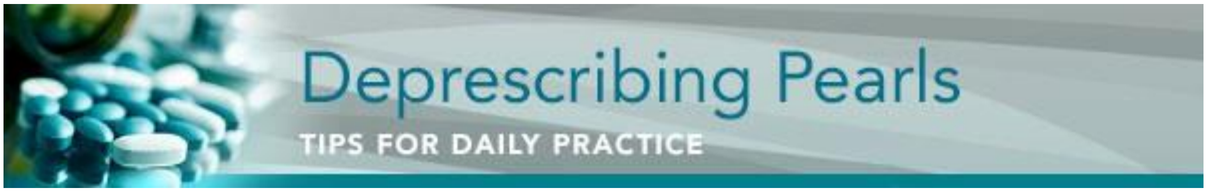

Skeletal Muscle Relaxants

Skeletal muscle relaxants are often risky for older adults:

- High-quality evidence indicates these medicines increase risk of falls and fractures in older adults.
- These medicines have anticholinergic properties (confusion, dry mouth, urinary retention, constipation) and may increase the risk of dementia.
- Many patients do not realize that these medicines work on the brain, NOT their muscles. Discussing this with patients can help them understand risks and increase motivation to deprescribe.
- These medicines are only recommended for short-term relief of acute musculoskeletal pain. Long-term use for pain should be avoided.

How to start the conversation with your patient:

- “I’m worried about you staying on your muscle relaxant. People who take these medicines are at higher risk of falling, memory problems, trouble urinating, feeling dizzy, dry mouth, and constipation. Have you experienced any of these?”
- “I have had a number of patients who’ve gotten off their muscle relaxant by trying other options, like topical treatments, for example. How about we look together at all the possible options and find the right alternative for you?”

Resources:

- KPWA clinical guideline for Non-specific Back Pain here.

STOP-FALLS Research Study

Email: Monica.M.Fujii@kp.org
Phone: 1-888-324-3166

KAISER PERMANENTE

UNIVERSITY of WASHINGTON

© 2024 Phelan EA et al. *JAMA Network Open.*

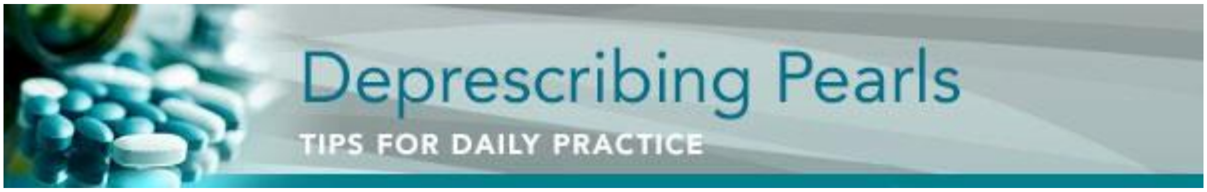

Tricyclic Antidepressants

Tricyclic antidepressants are risky for older adults:

- These medicines increase risk of falls, fractures, cognitive impairment, and urinary retention.
- Alternative treatments are available for neuropathic pain, insomnia, and depression and many have better risk-benefit profiles.
- Older adults may be taking these medicines along with others that also have anticholinergic properties. Anticholinergic burden due to concomitant use of such medications may adversely affect cognition and increase the risk of falls.

How to start the conversation with your patient:

- "I'm worried about you staying on [medication]. People who take this medicine are at risk of memory and concentration problems, not being able to urinate, feeling tired during the day, feeling dizzy, and traffic accidents. Have you experienced any of these?"
- "I have had a number of patients who've gotten off this medication by trying other options. How about we look together at all the possible options and find the right alternative for you?"

Resources:

- KPWA clinical guideline for Insomnia here.

STOP-FALLS Research Study

Email: Monica.M.Fujii@kp.org
Phone: 1-888-324-3166

KAISER PERMANENTE

UNIVERSITY of WASHINGTON

© 2024 Phelan EA et al. *JAMA Network Open*.

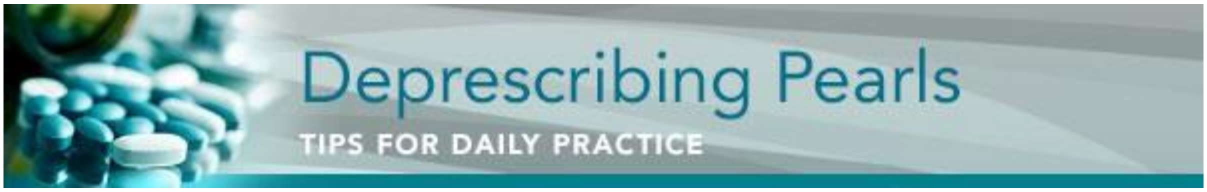

Managing Benzodiazepine and Z-Drug Withdrawal Symptoms

Many risky medications – including benzodiazepines and z-drugs – can be safely deprescribed in older adults:

- Recent guidelines recommend deprescribing of benzodiazepines and z-drugs to **all** older adults age 65+ who take these medications.
- Research has found that patients are more accepting of deprescribing if information is provided on what to expect and if there is a clear plan for tapering, including monitoring for return of underlying symptoms.
- Gradual taper of short-acting agents does not eliminate withdrawal symptoms but reduces their severity. When deciding on tapering doses and rates, consider using a slower rate with those with long-term use or history of psychological distress.
- Explain that it is common for a person to experience a brief (a few days to weeks) period of mild adverse drug withdrawal effects (e.g., insomnia, anxiety, restlessness) during tapering that will resolve with time.

How to start the conversation with your patient who is ready to taper:

- “If you are ready to slowly reduce the dose of your medicine, we can develop a plan together. My main priority is your safety and well-being. How does this sound?”
- “You might experience some short-term withdrawal symptoms, such as insomnia, anxiety, and restlessness. Any discomfort is usually temporary though. Are you interested in discussing a plan?”
- “We will do this in a stepped fashion. We can always go back to the previous step and resume tapering at a slower rate. Most patients are able to successfully reduce their dose or completely stop the medicine. How are you feeling about it today?”

Resources:

- KPWA clinical guidelines for:
  - Benzodiazepine and Z-Drug Safety here.
  - Insomnia here.

© 2024 Phelan EA et al. *JAMA Network Open.*

STOP-FALLS Research Study

Email: Monica.M.Fujii@kp.org
Phone: 1-888-324-3166

KAISER PERMANENTE

UNIVERSITY of WASHINGTON

© 2024 Phelan EA et al. *JAMA Network Open.*

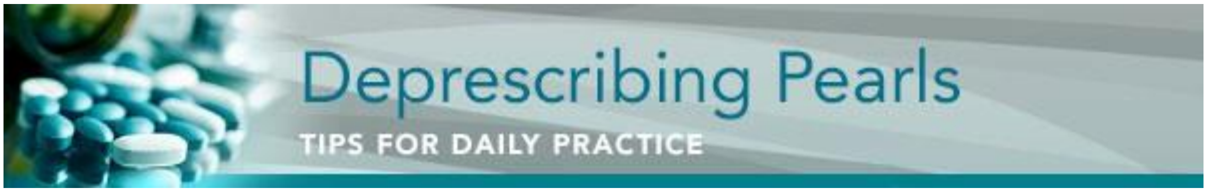

Fight Prescribing Inertia

Take a proactive approach when it comes to risky medications:

- Prescribing inertia is the tendency for medicines, once prescribed, to be continued for longer than they are safe or necessary.
- Older adults may have concerns that reducing or stopping a medicine may worsen their symptoms.
- Clear communication about the reason for stopping a medicine and the plan to address symptoms can reduce concerns.
- Focusing the conversation on safety concerns can reduce push-back.

How to start the conversation with your patient:

- “As we age, medicines that once were safe may no longer be safe. This is because our bodies process medicines differently at older ages. I want to make sure you are on the safest and most effective medicines. Is it okay if we review some of your medications today?”
- “Certain medicines can cause more side effects as we age, such as increasing the risk of falling. Let’s talk about your medicines.”
- “Although another doctor started this medicine, I want to make sure we are taking a fresh look at your medicine list now that some time has passed. Have you had any concerns or questions about your medications?”

STOP-FALLS Research Study

Email: Monica.M.Fujii@kp.org
Phone: 1-888-324-3166

KAISER PERMANENTE® UNIVERSITY of WASHINGTON

© 2024 Phelan EA et al. *JAMA Network Open.*

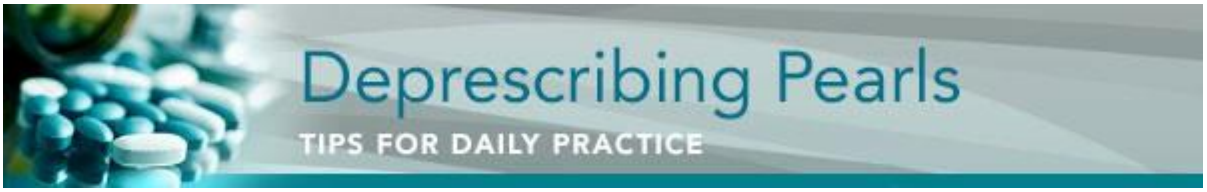

Pursuing Opportunities for Opioid Deprescribing

Patients often make statements (or "clues") during visits that suggest openness to non-opioid pain treatments or lower opioid doses:

- Some examples of "clues" are:
  - "[the medication] sometimes doesn't even work" [medication ineffective]
  - "with the higher strength, I get the brain fuzz that I don't like" [side effects]
  - "others have told me this medication is dangerous" [safety]
- Over half of patients on long-term opioid therapy endorse at least one side effect (most commonly constipation, sedation, and nausea) during their clinic visit. Systematically asking about side effects opens up opportunities to explore patient willingness to try alternative treatments or lower opioid doses.

How to start the conversation with your patient:

- "Many patients find that opioids don't work that well. I'd like to discuss if this medication is helping you feel less pain and do more. What are your thoughts about this?"
- "I hear your concerns about experiencing [opioid-related side effect]. [Opioid-related side effect] may be due to the [name of opioid] you are taking. It is a common side effect of this medication. Are you interested in trying to see if your side effect would get better if you tried a lower dose of [name of opioid], maybe combined with some other treatment options?"
- "I am glad you brought up safety concerns. Opioids do have a lot of risks; what have you heard?"

Resources:

- KPWA clinical guidelines for:
  - Chronic Opioid Therapy (COT) Safety for Patients on COT for Chronic Non-malignant Pain here.
  - Non-specific Back Pain here.

© 2024 Phelan EA et al. *JAMA Network Open*.

STOP-FALLS Research Study

Email: Monica.M.Fujii@kp.org
Phone: 1-888-324-3166

KAISER PERMANENTE
UNIVERSITY of WASHINGTON

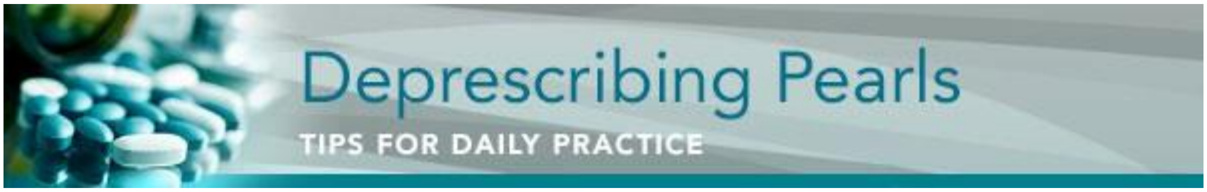

Deprescribing and the Patient-Provider Relationship

Providers may be hesitant to deprescribe for fear that it may damage their relationship with their patient. However, evidence suggests that deprescribing can occur without harming the patient-provider relationship:

- A majority of older adults are willing to reduce or stop their medicine if their provider recommends it.
- Evidence-based strategies to support better deprescribing discussions include:
  - Focusing on the benefits of taking fewer medications
  - Acknowledging that patients' need for and tolerance of medications may change over time; this may be particularly important for patients who are new to your practice
  - Making the connection between potential medication side effects and symptoms that a patient is experiencing (e.g., constipation, urinary retention, erectile dysfunction, memory problems, falls)

How to start the conversation with your patient:

- "You mentioned that you feel you are taking too many medications. I'm concerned as well. We can work together to review your medications and try to reduce the number you are taking over the next few visits. Shall we get started today?"
- "Our bodies change over time. This means that a medication that a person tolerated in the past could become less safe or start causing side effects. I'd like to review your medications and see if we can reduce any. How does this sound?"
- "You've expressed concerns about [symptom]. The [medication] that you are taking could be causing that. Would you be interested in trying to reduce the dose and perhaps finding an alternative to it?"

STOP-FALLS Research Study

Email: Monica.M.Fujii@kp.org
Phone: 1-888-324-3166

© 2024 Phelan EA et al. *JAMA Network Open.*

© 2024 Phelan EA et al. *JAMA Network Open.*

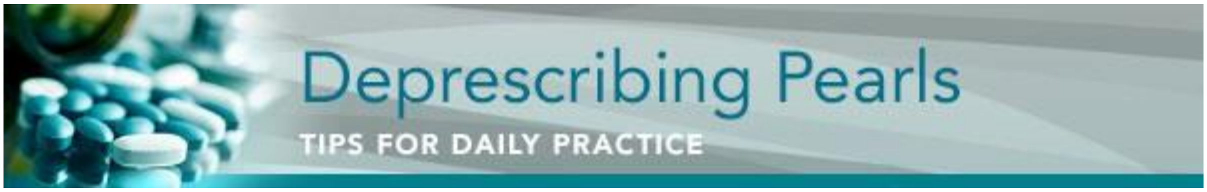

Return of Symptoms from Underlying Condition

During deprescribing, a patient may experience return of symptoms that the medicine was being used to treat:

- Patients often worry that their symptoms will recur and want to know that their provider will be accessible throughout the deprescribing process.
- Gradual tapering can minimize the impact of symptom recurrence and assist in identifying the lowest effective dose of a medication.
- It may help to recommend self-management strategies (e.g., sleep hygiene, physical therapy) at the same time as the taper so that the patient has tools to manage their symptoms.
- Ask the patient to track their symptoms (e.g., pain, depression, sleep, anxiety) using a log so that you have data to help guide the tapering process.

How to start the conversation with your patient:

- ""As we [reduce the dose of / stop] your medicine, I would like you to keep track of any symptoms and let me know as soon as possible if any do occur. We will work together to address them."
- "As you reduce this medication, you may notice [symptoms]. These usually go away with time. You may also notice some increase in your [main symptom that the medicine was being used to treat]. We can minimize this by starting some other approaches as we initiate the taper. Be patient with this process. In the end, I think you will be in a better place."

STOP-FALLS Research Study

Email: Monica.M.Fujii@kp.org
Phone: 1-888-324-3166

KAISER PERMANENTE® UNIVERSITY of WASHINGTON

© 2024 Phelan EA et al. *JAMA Network Open.*

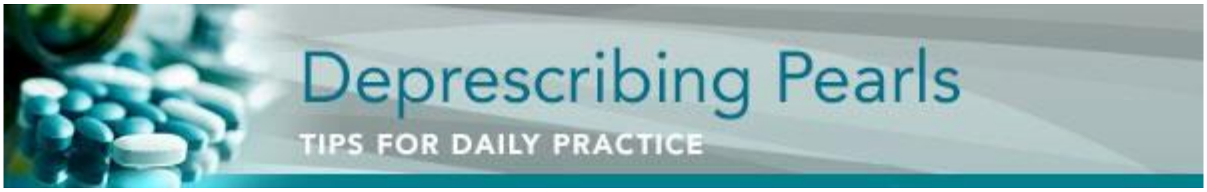

Deprescribing Triggers

Look for “triggers” or times when deprescribing is especially relevant to your patient:

- Successful deprescribing can be facilitated by identifying the right time to start the discussion, such as:
    - After a recent fall
    - If experiencing a symptom that may be a side effect of a medication, such as memory trouble, dizziness, or unsteadiness with walking.
- A recent systematic review found that medications that increase fall risk are not reduced following a fall-related healthcare encounter, suggesting a missed opportunity.

How to start the conversation with your patient:

- “I’m concerned about your use of [medication]. Your recent [injury – e.g., hip fracture] may have been due to [medication]. To help you avoid other injuries in the future, I’d like to discuss different treatment options.”
- “You are troubled by [symptom/complaint]. This may be a side effect of [medication] that you are taking. I recommend that we try to reduce the dose of that medication and see if your [symptom] improves. What are your thoughts about this?”

STOP-FALLS Research Study

Email: Monica.M.Fujii@kp.org
Phone: 1-888-324-3166

KAISER PERMANENTE

W UNIVERSITY of WASHINGTON

© 2024 Phelan EA et al. *JAMA Network Open.*

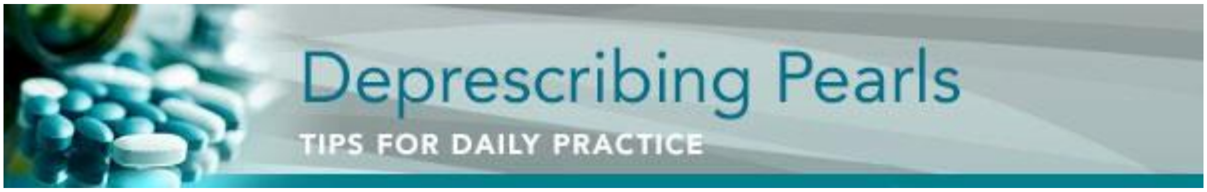

Deprescribing OTC Antihistamines

Antihistamine use is common, especially over-the-counter (OTC) antihistamines. Patients may not be aware of the risks associated with some OTC antihistamines (e.g., Benadryl), and their providers may be unaware that their patients are taking them. The 2019 American Geriatrics Society Beers List of potentially inappropriate medications recommends avoiding use of first-generation antihistamines in adults aged 65+. High-quality evidence indicates these medications increase risk of cognitive impairment, delirium, dementia, and urinary retention.

The STOP-FALLS study seeks to help older adults reduce use of medications that act on the central nervous system and may increase fall risk. As part of this study, participating patients receive an educational brochure on first-generation antihistamines which describes medication risks and alternative strategies for managing their symptoms, including self-help tips for symptom management. The brochure also encourages patients to have conversations with their providers about their use of these medications.

As a provider at a participating clinic, we are sending you a link to an Evidence-Based Pharmaceutical Opinion (EBPO) for antihistamines. The EBPO describes risks associated with these medications, tapering suggestions, and alternative treatments. If your patient wants to have a conversation with you about their antihistamine use, we hope the EBPO will facilitate your conversation. The guidance provided in this EBPO is a recommendation, but your clinical judgment is best, and there is no action that is required on your part. All patient and provider materials were developed to align with KPWA guidelines and reviewed and approved by KPWA pharmacy and physician leadership.

To view the EBPO for first-generation antihistamines, please visit:
www.kpwashingtonresearch.org/stopfalls/antihistamines

And to learn more about the STOP-FALLS research study please visit:
www.kpwashingtonresearch.org/stopfalls/overview

STOP-FALLS Research Study

Email: Monica.M.Fujii@kp.org
Phone: 1-888-324-3166

KAISER PERMANENTE® UNIVERSITY of WASHINGTON

© 2024 Phelan EA et al. *JAMA Network Open.*
