# Supplementary material for: Reducing Central Nervous System–Active Medications to Prevent Falls and Injuries Among Older Adults: A Cluster Randomized Clinical Trial
Source: JAMA Netw Open. 2024 Jul 25;7(7):e2424234. doi: 10.1001/jamanetworkopen.2024.24234 (PMC11273227; doi:10.1001/jamanetworkopen.2024.24234)
Supplement: Supplement 3. — Data Sharing Statement [file jamanetwopen-e2424234-s003.pdf]

Data Sharing Statement

Phelan. Reducing Central Nervous System–Active Medications to Prevent Falls and Injuries Among Older Adults. *JAMA Netw Open*. Published July 25, 2024.
doi:10.1001/jamanetworkopen.2024.24234

Data
Data available: No
